# Experimental determination of the oral bioavailability and bioaccessibility of lead particles

**DOI:** 10.1186/1752-153X-6-138

**Published:** 2012-11-22

**Authors:** Elise Deshommes, Robert Tardif, Marc Edwards, Sébastien Sauvé, Michèle Prévost

**Affiliations:** 1 Civil, Geological, and Mining Engineering Department, NSERC Industrial Chair on Drinking Water, Ecole Polytechnique de Montréal, CP 6079, Succ. Centre-Ville, Montréal, QC, H3C 3A7, Canada; 2 Environmental Health and Occupational Health Department, ESPUM (École de Santé Publique de l’Université de Montréal), C.P. 6128, succ. Centre-ville, Montréal, QC, H3C 3J7, Canada; 3 Department of Civil and Environmental Engineering, Virginia Tech University, 418 Durham Hall, Blacksburg, VA, 24061, USA; 4 Department of Chemistry, Université de Montréal, 2900, Édouard-Montpetit, Montréal, QC H3C 3A7, Canada

**Keywords:** Oral bioavailability, Bioaccessibility, Lead [Pb] particles, *In vivo* tests, *In vitro* tests

## Abstract

*In vivo* estimations of Pb particle bioavailability are costly and variable, because of the nature of animal assays. The most feasible alternative for increasing the number of investigations carried out on Pb particle bioavailability is *in vitro* testing. This testing method requires calibration using *in vivo* data on an adapted animal model, so that the results will be valid for childhood exposure assessment. Also, the test results must be reproducible within and between laboratories. The Relative Bioaccessibility Leaching Procedure, which is calibrated with *in vivo* data on soils, presents the highest degree of validation and simplicity. This method could be applied to Pb particles, including those in paint and dust, and those in drinking water systems, which although relevant, have been poorly investigated up to now for childhood exposure assessment.

## Review

### Introduction

Lead [Pb] is hazardous to humans, and is of particular concern for fetuses, infants, children, and women of child-bearing age [1,2]. Knowledge of Pb toxicity has progressed significantly since the 1970s. Initially considered toxic only at blood lead levels [BLLs] over 60 μg/dL, it is now considered to have no definable threshold below which “no harmful effect” can be determined [3]. Early studies demonstrate the immediate and long-term effects of low Pb level exposure during childhood, such as neurobehavioral and neurodevelopmental deficiencies, and effects on growth, hearing, and blood pressure [3,4]. Pb uptake may reduce the intelligence quotient [IQ] of infants and children, and affect some brain responses, even at low BLLs. Recently, the effect on IQ has been shown to be even stronger at low BLLs, since decrements in IQ were more dramatic between BLLs categories < 5 μg/dL and 5–9.9 μg/dL than between categories 5–9.9 μg/dL and ≥ 10 μg/dL, which has led to a reconsideration of the 10 μg/dL health advisory level [5-8]. Indeed, the European Food and Safety Agency (EFSA) has set the 95^th^ percentile lower confidence limit of the benchmark dose [BMD] of an incremental 1% risk (BMDL01) at 1.2 μg Pb/dL as a reference point when assessing the risk of intellectual deficits in children measured by the Full Scale IQ score [9]. Also, the EU Risk Assessment Report states that Pb exposure effects cannot be measured reliably at BLLs < 5 μg/dL, due to the limited precision of methods of behavioral testing and Pb quantitation in blood [10]. Considering the neurocognitive and neurophysiological effects observed at low BLLs, a new reference level of 5 μg/dL was recently set by the CDC, and will be revised downwards every 4 years [11].

Early studies on the health impacts on children led to public policies to remove Pb sources from food and leaded gasoline, resulting in the steady decline of BLLs in recent years [3,12-15]. Remedial actions have also been taken to decrease Pb levels in the soil and dust of Pb-contaminated sites, although decreasing these levels may not always result in lower BLLs in children [14-17]. Lead paint is a common cause of clinical Pb poisoning, and remains a strong contributor to childhood exposure, as it is still present in houses built before 1978 in the United States; its abatement cost is estimated in the billions of dollars [3,14,15]. Now, as BLL guidelines become more stringent, the relative contribution of tap water to total Pb exposure may become highly significant [18-21]. Lanphear et al. report that Pb occurrence in water > 5 μg/L after 1 min of flushing increases the BLLs of children aged 6–24 months by 1.02 μg/dL, contributing to about 20.4% of the total BLL [22]. Recently, abnormal BLLs in young children have been associated with elevated Pb concentrations in drinking water, particulate Pb representing a significant fraction of this [23-26]. However, because of its sporadic occurrence, exposure to particulate Pb in drinking water is especially difficult to characterize [27]. Most regulatory and research efforts have assumed that soluble Pb is the predominant form of exposure. Interest in particulate Pb is now growing, because of its potential contribution to chronic and acute exposure [27-29]. The most direct approach to assessing the contribution of various Pb sources to human exposure is to conduct epidemiological studies or *in vivo* studies. Numerous epidemiological studies of particulate Pb exposure from various media (i.e. air, soil, paint, and dust) have been reviewed [14,15,30,31]. Some of these revealed a significant relationship between environmental Pb levels and BLLs, and a decrease in BLLs after remediating Pb sources, providing direct measurements of exposure and its impacts on body burden. However, they are challenging to carry out because of cost, the variability of exposure among the population studied, and BLL evolution since exposure [32].

*In vivo* experiments are much simpler to plan and perform than epidemiological studies. Key parameters can be controlled, such as exposure levels and conditions, and the relationships between exposure levels and levels of Pb absorbed by the *in vivo* specimen tested can be quantified. However, the measurements are indirect, based on animal models, and require extrapolation to human exposure conditions. A primary outcome of *in vivo* studies of oral Pb exposure is the oral bioavailability of Pb occurring in both dissolved and particulate form. Several *in vivo* experiments have been performed, first with readily soluble Pb forms such as Pb acetate [PbAc], and then mostly with Pb-contaminated soils. But comprehensive bioavailability experiments are long, expensive, demanding, and the use of animal models can raise ethical issues. Moreover, bioavailability results show significant variability, which is inherent to the use of animal models.

*In vitro* experiments are simpler, faster, cheaper, and highly reproducible, and do not raise ethical concerns, and therefore are best suited to test replication. However, they can only assess the bioaccessibility of Pb, defined as the fraction of ingested Pb dissolved during the digestion process and available for absorption into the systemic circulation (bloodstream). Bioavailability is the fraction actually available and taken up by an organism, while bioaccessibility is an experimentally determined estimate of what is potentially bioavailable [33]. If well calibrated, an *in vitro* test can adequately predict *in vivo* results. A significant number of bioaccessibility studies have been conducted on Pb contaminated soils. However, the bioaccessibility of other sources of Pb particles to which infants/children can be exposed (paint, dust, drinking water particles, toys, and food) has not been thoroughly investigated. *In vivo* testing of these particulate forms can be limited by experimental constraints, which can be addressed using *in vitro* tests.

This review summarizes the information available on the bioavailability and bioaccessibility testing that have been proposed for estimating the oral bioavailability of Pb particles in children. Our objectives are to identify the strengths and weaknesses of these procedures, to highlight differences between them to support the interpretation of study conclusions, and, finally, to draw attention to gaps in the data on Pb particles that are relevant for childhood exposure assessment.

### Approaches to estimating Pb bioavailability

Many definitions of bioavailability have been proposed for soil and sediments [33]. In this review, the term “oral bioavailability” refers to the amount of ingested Pb that reaches the systemic circulation and that is likely to accumulate in the body, including organs and bones. Historically, bioavailability has been determined in the laboratory using *in vivo* testing. *In vivo* testing to estimate Pb bioavailability must be carried out over an extended period to ensure that the metal is absorbed, retained, and excreted. The half-life of Pb is about 25 days in blood, 40 days in soft tissues, and 25 years in bones [34]. Early human experiments were conducted using Pb in the diet, Pb tracers, or Pb salts labeled with radioactive Pb^203^[35-37]. A detailed review and critical evaluation of these studies is available in Mushak (1991) [30]. Such studies helped identify absorption mechanisms for soluble Pb and interactions with food, although there are still some uncertainties on the specific sites where Pb uptake actually takes place [30]. However, these results cannot be directly extrapolated to Pb particles in soil, dust, paint, or drinking water, because of potential matrix effects. A more recent study measured the bioavailability of soil borne Pb in human adults, and is the only study performed on humans with Pb-contaminated soil [38]. The target population was adults, whose digestive absorption processes differ markedly from those of infants and children [30].

Most *in vivo* experiments have been conducted with young animals using various experimental designs. In general, several groups of animals are involved: a control group receiving a purified diet without any Pb; a group receiving a Pb salt-based solution, such as PbAc, either orally or intravenously (or both, if there are two groups), at different dosages representing the Pb dose range of the test materials; and, finally, a group administered with different doses of the test materials. Similar Pb doses from the test materials and from PbAc are necessary to ensure that the slopes of dose–response curves for both materials are comparable. Also, the range of the dosages applied usually includes low to high doses so that some degree of active transport (saturable) is represented in the blood compartment. Pb salts such as PbAc (soluble) are used as references to compare test materials and calculate bioavailability. Administering Pb salts intravenously allows the absolute bioavailability [ABA] – the fraction of Pb that enters the bloodstream (absorption fraction) – to be calculated (Equations 12). If PbAc is delivered orally, the relative bioavailability [RBA] – the bioavailability of the test material relative to that of PbAc – is calculated (Equation 3). The intravenous dosage for determining ABA is also used to set minimum and maximum BLLs for a range of doses and to better characterize Pb distribution in a steady state [39-41]. RBA is also called “oral bioavailability”, but relative to an appropriate reference material (PbAc) [41,42]. RBA is more informative, because it takes the exposure matrix into account [43]. The ABA can be calculated, as opposed to measured, through intravenous injections, by multiplying the RBA by the Pb absorption fraction (ABA) of PbAc administered orally (Equation 4).

Oral ABA calculation:

(1) $$ABA_{\mathrm{TM}}=\frac{IDM_{oral\;TM}}{IDM_{iv\;PbAc}}\times \frac{Dose_{iv\;PbAc}}{Dose_{oral\;TM}}\text{.}$$

(2) $$ABA_{\mathrm{PbAc}}=\frac{IDM_{oral\;PbAc}}{IDM_{iv\;PbAc}}\times \frac{Dose_{iv\;PbAc}}{Dose_{oral\;PbAc}}\text{.}$$

Oral RBA calculation:

(3) $$\;RBA_{\mathrm{TM}}=\frac{ABA_{\mathrm{TM}}}{ABA_{oral\;PbAc}}\text{.}$$

$$RBA_{\mathrm{TM}}=\frac{IDM_{oral\;TM}}{IDM_{oral\;PbAc}}\times \frac{Dose_{oral\;PbAc}}{Dose_{oral\;TM}}\text{.}$$

Link between ABA and RBA:

(4) $$ABA_{\mathrm{TM}}=ABA_{oral\;PbAc}\times RBA_{\mathrm{TM}}\text{.}$$

IDM – Internal Dose Metrics (BLL, Pb in tissues, etc.); iv – intravenous; TM – Test Material.

The internal dose metrics [IDM] measured during *in vivo* experiments can also vary. Several kinetic pools for Pb in the human body with varying rates of turnover, partly depending on the time elapsed since the administration of the Pb dose, affect bioavailability estimation. To address this, sequential blood samples can be collected during the experiment, as well as blood and tissue samples on the last day of the test. The tissue samples most frequently collected are from the liver, kidneys, and femurs. The amount of Pb stored in other parts of the body is considered to be small, in the order of 4% of total Pb measured in blood, bones, liver, and kidneys for short-term experiments [44]. So, RBA calculation can be based on Pb levels either in blood or in tissues: blood-based RBA is calculated by measuring the ratio between BLL following test material ingestion and BLL following oral ingestion of PbAc for a similar dosage (Equation 3). This ratio is measured under presumed steady state conditions after repeated exposure, resulting in a stable BLL, or at various times during the experiment to monitor BLL evolution [44]. Similar ratios can be established for bones, kidneys, and liver on the last day of the experiment [45-47].

**Figure 1:**
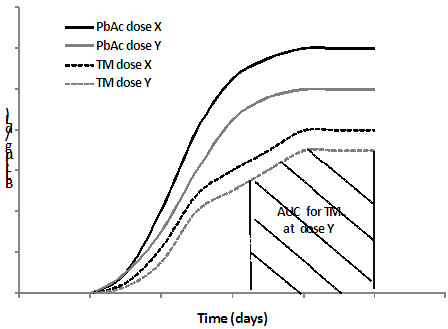
**Calculation of blood Area Under the Curve (AUC). ***TM – Test Material.*

**Figure 2:**
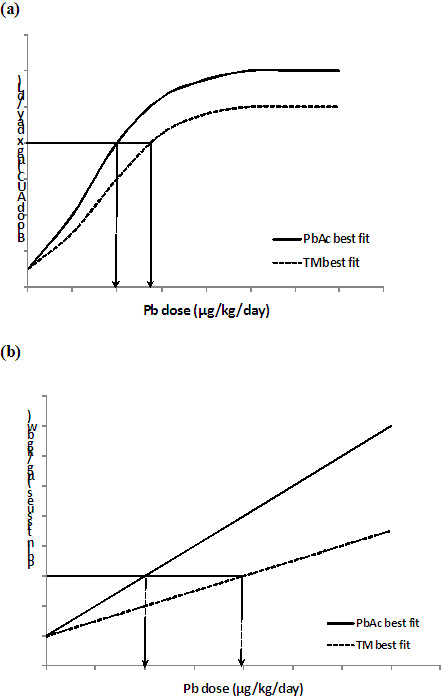
**Calculation of RBA based on equivalent Internal Dose Metrics (IDM) for (a) blood; and (b) tissues (bones, liver, kidney, etc.). ***TM – Test Material.*

The most commonly used approach for evaluating bioavailability, and a comprehensive one, is based on monitoring BLLs over the course of the experiment for different dosages, as well as the Pb content in bones, kidneys, and liver at the end of the exposure period for different dosages. The “area-under-the-blood concentration versus time curve” [AUC] of BLL is then calculated for each Pb dose and test material (Figure 1). The AUC approach for BLL has the advantage of including the whole dose–response relationship of the BLL over the time of the experiment. However, the information it provides on the evolution of absorption over that period is poor [48]. AUC BLL (e.g. μg×day/dL), bone Pb levels, or other kinds of IDM for Pb salt and test material are then fitted versus Pb dose. Identical absorbed doses of Pb delivered to target Pb pools are assumed to produce an equivalent IDM [40]. Bioavailability is determined by calculating the ratio of Pb test material dose and the PbAc dose that yields an identical IDM [40,49] (Figure 2). This calculation is equivalent to Equation 3, since the IDM for both the test material and the PbAc administered orally cancel each other out:

Oral RBA based on equivalent IDM:

(5) $$RBA_{\mathrm{TM}}=\frac{\text{Dos}e_{\text{PbAc}\;\text{oral}\;\text{producing}\;\text{equivalent}\;\text{IDM}\;\text{to}\;\text{TM}}}{\text{Dos}e_{\text{TM}\;\text{oral}\;\text{producing}\;\text{equivalent}\;\text{IDM}\;\text{to}\;PbAc}}\text{.}$$

This approach requires finding the most suitable mathematical model for both the PbAc dose and the Pb test material dose IDMs. Indeed, an IDM per unit of absorbed Pb dose does not depend on the Pb source. The best fit for bone, liver, and kidney Pb levels is usually linear, whereas for BLL/AUC, which increases gradually and then reaches a plateau (steady state), the best fit is non linear and varies [42,49]. Doses of Pb test material and Pb salt producing the same IDM can be calculated from the best fitting equations, and the bioavailability ratio calculated for each endpoint studied (AUC, BLL, Pb in bones, etc.). The BLL/AUC used for this ratio is usually the point before the rate of uptake of Pb from the test material starts to decrease owing to increasing dosage [40]. The ratio can also be calculated for a range of doses that reflects currently estimated exposure. Finally, a point estimate can also be calculated, which combines the bioavailability related to each of the endpoints studied simultaneously, and is weighted according to its uncertainty. However no significant differences were shown between using only a BLL estimate or a point estimate based on multiple IDM [42,49].

Another way to evaluate bioavailability is to complete a total mass balance. This method requires sequential BLL sampling, as well as total collection of urine and feces to recover all the excreted Pb. Pb content in feces and urine is subtracted from total Pb intake, and provides an estimate of the body’s net retention [37]. Urine samples represent the absorbed dose of Pb that is primarily excreted, whereas fecal samples provide the fraction of Pb that is unabsorbed [50]. When BLLs reach a steady-state with multiple dosing, bioavailability can be determined by calculating the ratio of *total Pb in the urine plus total Pb retained in the body* to *total Pb recovered*. Such results have limited value if the total net excretions are underestimated. However, this method is nearly non invasive for the test subject, and can provide information on Pb accumulation in the body after repeated exposure. This makes it useful for studies on Pb uptake and Pb distribution in the whole body [48].

The method for calculating the bioavailability and IDM on which it is based directly influences the bioavailability estimates. Understanding these differences is important when comparing results based on different methodologies. An RBA based on BLL is unlikely to provide the same results as global bioavailability based on the whole body’s Pb uptake derived from a complete mass balance. If bioavailability is based on the blood AUC, the results will be influenced by the duration of the experiment and the timing of sampling. Furthermore, the interactions between Pb in soft tissues, bones, and blood determine the fractional absorption, and thus the bioavailability [51]. The timing of the sampling should be chosen carefully, considering the evolution of BLL over time and whether or not steady state is reached [52].

### Experimental factors influencing Pb bioavailability

The *in vivo* experimental design parameters affecting the estimation of Pb bioavailability include: the animal model (species), its age, the addition of food, the duration of the experiment, and the dosage. The selection of these parameters and corresponding bioavailability results for published studies are summarized in Table 1.

**Table 1:** ***In vivo *experiments on Pb particles and bioavailability results (2 pages)**

**REFERENCE**	**SPECIMEN**	**SUBSTRATE**	**STATE, DURATION, DOSE**	**RBA/ABA**
**Freeman et al. 1992**[45]	Rats (7–8 wk)	Mine wastes	· Fed	· 12.1±3.6-26.8±4.8 (blood RBA %)
			· 30 d	· 4.8±1.9-13.3±2.2 (bone RBA %)
			· 0.12-24 mg Pb/kg bw/d	· 0.6±3.1-13.6±3.1 (liver RBA %)
**Dieter et al. 1993**[53]	Rats (6–7 wk)	Pb oxide, Pb sulfide, Pb ore concentrate	· Fed	· 69-93 (blood RBA %, Pb oxide)
			· 30 d	· ND-36 (blood RBA %, Pb sulfide)
			· 0–100 ppm Pb	· ND-10 (blood RBA %, Pb ore)
**Freeman et al. 1993**[54]	Rats (7–8 wk)	Mine wastes	· Fed	· 0.36±1.04-7.32±1.57 (blood ABA %)
			· 30 d	· 0.51±0.15-2.25±0.23 (bone ABA %)
			· 5 to 20 ppm Pb	
**Ruby et al. 1993**[55]	Rabbits (12 wk)	Mine wastes	· Fast	· 9±4 % Pb soluble in stomach
			· 0.5-36 h	
			· 7.8 mg Pb/kg bw	
**Freeman et al. 1994**[39]	Rats (7–8 wk)	Mine wastes	· Fed	· 2.7±1.5 (% blood RBA)
			· 30 d	· 0.40±0.16 (% bone RBA)
			· 0.12 to 24 mg Pb/kg bw/d	· 0.55±0.68 (% liver RBA)
**Schoof et al. 1995**[40]	Rats (4 wk)	Smelter soil	· Fed	· 41 (% blood RBA)
			· 31 d	· 20 (% blood ABA)
			· 0.11-3.4 mg Pb/kg bw/d	
**Freeman et al. 1996**[44]	Rats (~ 4 wk)	Soil, Pb sulfide	· Fed	· 0.8-8.7 (soil, % RBA)
			· 44 d	· 1.2-5 (Pb sulfide,% RBA)
			· 17.6-127 ppm	
**Lorenzana et al. 1996**[56]	Swine (40–50 d)	Tacoma smelter soil & slag	· Fast (small dough)	· Mean ABA^§^ (%, PbAc_iv_): 10 (soil), 4 (slag)
			· 0–7 d	
			· 34–567 μg Pb/kg bw (single dose)	
**Casteel et al. 1997**[49]	Swine (8–9 kg)	Berm and residential soils	· Fast (small dough)	· 74-75 (point estimate RBA %^‡^)
			· 15 d	
			· 71–732 μg Pb/kg bw/d	
**Casteel et al. 1997-1998**^**#**^	Swine	Joplin smelter soil treated or not with 1% phosphate		· Point estimate RBA (%): 59–67 (not treated) to 38–45 (treated 1% phosphate)
**Maddaloni et al. 1998**[38]	Humans (21–40 yr)	Bunker Hill residential soil	· Fast & Fed	· Fasting state: 26.2±8.1 (% ABA)
			· 30 h	· With breakfast meal: 2.52±1.7 (% ABA)
			· 250 μg Pb/70 kg bw	
**Ellickson et al. 2001**[57]	Rats (0.18–0.2 kg)	SRM 2710 Montana soil	· Fast	· 0.4-0.9 (% RBA)
			· 3 d	
			· 7–8 mg Pb (single dose)	
**Brown et al. 2003**[58]	Rats (3–4 wk)	Urban soil treated with biosolids (n=9)	· Fed	· RBA not calculated in %
			· 35 d	
			· 71–125 mg Pb/kg diet	
**Hettiarachchi et al. 2003**[59]	Rats (12 wk)	Joplin soil treated or not with Mn, phosphate, or CRYP (n=15)	· Fed · 21 d	· Blood RBA%: 34 (not treated); 19–33 (treated)
			· 1–6 mg Pb/kg bw/d	· Kidney RBA%: 48 (not treated); 19–39 (treated)
				· Liver RBA%: 27 (not treated); 19–21 (treated)
				· Bone RBA%: 34 (not treated); 20–24 (treated)
**USEPA 2004, 2009**[60,61]	Juvenile swine	2 Omaha smelter soils	· Fast (small dough)	· 83 & 96 (point estimate RBA %)
			· 15 d	
			· 75–675 μg Pb/kg bw/d	
**Casteel et al. 2006**[42]	Swine (5–6 wk)	19 soil or soil-like materials^†^	· Fast (small dough)	· 1-105 (point estimate RBA %^*^)
			· 15 d	
			· 75–675 μg Pb/kg bw/d	
**Marschner et al. 2006**[62]	Swine (70 d)	5 soils	· Semi-fed (milk powder after 5 h fast)	· 17-63 (RBA %)
			· 28 d	
			· 0.1-3.2 mg Pb/kg bw/d	
**MSE Technology Application 2006**[63]	Swine (5–6 wk)	Smelter soil HER-2930	· Fast (small dough)	· 82 (point estimate RBA %)
			· 15 d	
			· 77–686 μg Pb/kg bw/d	
**Smith Jr. et al. 2008**[46]	Rats (~ 21 d) Micropigs (~ 30 d)	2 smelter soils	· Fed	· Rats RBA %: 88 (blood), 62 (bone)
			· 30 d	· Micropigs RBA %: 81 (blood), 68 (bone)
			· 50 μg Pb/g diet	
**Smith Jr. et al. 2008**[47]	Rats (~ 21 d)	5 soils	· Fed	· 85±48 (blood RBA %)
			· 35 d	· 91±12 (bone RBA %)
			· 6.8-150 μg Pb/g diet	
**Bannon et al. 2009**[64]	Juvenile swine	8 small arms range soils	· Fast (small dough)	· 108±18 (point estimate RBA %)
			· 15 d	
			· 75–675 μg Pb/kg bw/d	
**Caboche 2009 Denys et al. 2012**[65,66]	Swine (28 d)	15 mining and smelting soils	· Fast (small dough)	· 6-100 (kidney RBA %)
			· 14 d	· 8–100 (urine RBA %)
			· <1 to 8 mg Pb/kg bw/d	· 9–100 (bone RBA %)
				· 10–85 (liver RBA %)
**Juhasz et al. 2009**[67]	Swine (6–8 wk)	5 incinerator and urban soils	· Fast	· 10.1±8.7-19.1±14.9 (blood RBA %)
			· 5 d	
			· 0.1-1.2 mg Pb/kg bw (single dose)	
**Smith et al. 2011**[68]	Adult mice	12 Pb impacted soils from various sources	· Fast	· 10±2.8-89±15.3 (blood RBA %)
			· 48 h	
			· 0.1-1.7 mg Pb (single dose)	

RBA–relative bioavailability; ABA–absolute bioavailability; ND–not determined; ^§^estimates include 0 & 100% in the confidence interval; ^‡^USEPA and Casteel et al. point estimate [42,43]; ^#^data from the Ruby et al. review [69]; ^†^including soils tested by Schroder et al. [70]; ^*^also available in USEPA [43].

#### Animal model

A major factor affecting bioavailability estimation is the animal species used, and includes its age and its developmental stage. Intra-species differences in anatomy, feeding behavior, absorption rate, and digestion processes influence the results. As shown in Table 1, rats, piglets, and rabbits have been used to estimate Pb particle bioavailability. Pioneer studies used rodents and rabbits to determine bioavailability and the risk to infants/children [39,44,45,54,55,71-74].

Although rodents and lagomorphs are widely used and well-known animal *in vivo* models, differences in their digestive systems compared to the human prevent direct extrapolation to the Pb exposure of human children. First, rodent stomachs have a smaller glandular region than human stomachs, and consequently less surface area for parietal cells secreting acid [48]. This results in a higher stomach pH of 3.9 and 3.2 in the fasting and fed states respectively [75]. In contrast, rabbits present a relatively low pH of 1.6 in the fed state, which is significantly lower than that of humans [76]. Also, the surface area of the small intestine of a rat is about one-fifth the size a human one, which implies decreased Pb bioavailability, since some absorption mechanisms in the small intestine are surface area-dependent [48]. Another significant difference is that the rat’s small intestine is mature at weaning, unlike that of the human newborn, and its Ca absorption capacity decreases rapidly within 30 days [48]. This results in a drastic decrease in the rat’s Pb absorption rate, from about 80% between 0 and 30 days to about 50% between 1–2 weeks and 6–8 weeks [30,51]. Several *in vivo* studies listed in Table 1 have used rats aged 4 weeks and 6–7 weeks [39,40,44,45,54,57], which may have been too old to be representative of the high Pb absorption rates found in children 0–6 years old [52]. Differences in the soils tested aside, the animal’s age may actually explain the significantly higher blood RBA (85-88%) observed in weaning rats aged 21 days [46,47], compared to that of rats aged 4 weeks and older (0.8-41%) [39,40,45,54]. Also, the 12-week old rabbits used by Ruby et al. [55] may be more representative of adult digestive conditions, as a 5-week old rabbit absorbs twice as much Pb as an older rabbit [52]. The declining absorption rates within a relatively short period constitute a challenge in itself, since bioavailability tests must extend over several weeks owing to the half-life of Pb in blood. During a subchronic testing period of 15–30 days, the weanling rat’s Pb absorption can vary significantly, depending on its age at the start of the testing period.

Specific behavior and defense mechanisms of rats and rabbits also influence absorption. For example, in response to exposure to Pb, rats lower their body temperature, which decreases its RBA. Furthermore, the constant feeding pattern of rats and rabbits and their cellulose-type diet are not favorable to Pb dissolution in the stomach, as this organ rarely empties completely, resulting in a pH buffer effect and the constant availability of ligands for Pb ions [48]. Finally, coprophagic behavior in both species can lead to an inaccurate estimation of Pb bioavailability, because of Pb recirculation. Coprophagy is essential to the assimilation of all the nutrients necessary for the growth and development of rodents and rabbits, which, if impeded, can cause deficiencies in some minerals/vitamins that interfere with Pb absorption. Biliary excretion is also greater in rodents than in humans, and can generate errors in the estimation of Pb excreted and absorbed [40,46-48]. Despite these differences, rats and rabbits are species commonly used for *in vivo* experiments, and so are very well characterized. Interpretation of the results, adjustments to them, and extrapolation to humans may therefore be more accurate than for other animal models that are less often used in laboratories. Moreover, even with the marked differences between their digestive systems and those of children, these species remain highly useful for studying many aspects of Pb exposure, such as estimating the potential health risks of Pb (e.g., [77]).

Smith Jr. et al. compared Pb bioavailability in weanling micropigs and rats for various test materials, concluding that the micropig model is superior, because of a greater Pb concentration in their tissues and the ability to detect bioavailability trends in their bones, blood, liver, and kidneys [46]. Juvenile swine appear to be a better surrogate for predicting digestive and absorption processes in infants, because of the similarities between them with respect to: gastric hydrochloric acid (HCl) and protease secretion; small intestine configuration; limited digestive capacity, which impedes solid food digestion; and gut maturity [34,78]. At birth, the digestive organs of both species are comparable in size, and the anatomy of the stomach and small intestine is similar. Also, although the immature swine grows faster than the newborn/child, it remains at prepubertal state throughout the experiment [41]. Similar patterns for secretions of HCl, pepsin, and other enzymes are present in the stomach, although gland distribution differs. Stomach capacities for babies at birth, 2 weeks, and 4 weeks approach piglet stomach volumes at 0, 10, and 20 days. Finally, the length of the small intestine and the microscopic gut structure of infants and juvenile swine are similar [78]. Marked differences also exist in the capacity of the digestive tract relative to the body weight of the piglet, which is three times greater than the newborn’s. The piglet’s stomach volume is 260 cm^3^, compared to 130 cm^3^ for infants of the same weight (5.75 kg). In the piglet, the length of the small intestine increases dramatically within the first ten days of life compared to that of the newborn. This implies that adjustments need to be made, notably in terms of increases in feed levels/dosage. However, the piglet’s gastro-intestinal [GI] tract is well characterized, and the relationships between body weight and stomach/small intestine weight/length are known, so that adequate adjustments can be made. To account for the fact that the juvenile swine grows seven times faster than the human baby, intake is expressed relative to gut capacity at similar developmental stages. This rapid growth can be an advantage, since it provides an accelerated model for postnatal human growth and development. Finally, the piglet’s size allows serial blood sampling without stressing the animal [78]. However, the cost of conducting piglet experiments can limit the use of this model to investigating multiple substrates.

USEPA Region VIII scientists conducted *in vivo* experiments on piglets to estimate the bioavailability of soil Pb particles, and from there the potential burden for infants/children [41,79,80]. The juvenile swine model experimental procedure for assessing oral bioavailability from soils, detailed in Weis et al. [41] and further developed by Casteel et al. [42], was then applied to a wide variety of soils [60,63,64,70], in some cases with modifications to the original EPA procedure [46,62].

#### Conditions of dose and application

The dosage and its frequency are key factors affecting bioavailability estimation [41]. Generally, a fixed quantity of soil/dust is administered based on the estimated average daily soil/dust intake for children, which corresponds to a variable Pb dosage depending on the Pb content of the soil. However, for some animal models, such as the rat, it is often necessary to increase dosages in order to increase BLLs to detectable levels that permit reliable differentiation between the PbAc and the test material [45]. Bioavailability results obtained under such conditions may be underestimated, as the rate of elimination of Pb at these high dosages could be much higher, especially if they largely exceed the saturation concentration for active transport mechanisms in the gut [79].

Childhood soil/dust ingestion rates from hand-to-mouth activity refer to small quantities ingested repeatedly during the day, resulting in a cumulative dose of around 100 mg/day [52]. The bioavailability estimation based on *in vivo* experiments is only valuable if the ingestion of the test material/PbAc and subsequent absorption by the test animal are representative of those of children ingesting Pb from diet, soil, dust, paint, or water [48,52]. Mushak’s review stresses that the dosages for *in vivo* testing should be representative of children’s exposure in terms of amount and number of ingestions [52]. For example, the single high dose of 4.2 g of test material in the Ruby et al. study [55] is not representative of normal children exposure, but more akin to soil geophagia, a form of pica ingestion [52]. Realistic exposures can be achieved by administering lower doses twice daily, as performed in several swine studies in Table 1[42,49,60,61,63-65]. These studies suggest that realistic dosages administered through multiple daily ingestions are preferable, and represent a better simulation of the exposure of the infant/child.

The choice of a fasting state or a fed state when administering Pb doses is a major factor in calculating bioavailability, since the stomach’s retention time and pH, which are determining factors for Pb dissolution, depend on that state. *In vivo* studies presented in Table 1 were performed either in fasting or fed state. The uptake of PbAc is reduced by about half when Pb is administered in food, rather than in a fasting state as in rat-based experimental studies [43]. Maddaloni et al. also observed a decrease in RBA of about 24% from fasting to feeding conditions in human adults after ingesting contaminated soil [38]. The type of food also significantly influences bioavailability, as shown by the wide retention range associated with food type (3.5-56.8%), as compared to 61.3% for fasting adults dosed with PbAc [35]. That study concluded that Pb ingestion during meals, as well as the presence of calcium, phosphate, and phytate in the meals, notably decreases Pb bioavailability three hours before and after ingestion. Indeed, calcium, iron, and phosphate were shown to compete with Pb for absorption through the intestine because of similar uptake mechanisms, although Pb uptake was not evidenced to occur via active iron transporters or calcium channels [30,34]. Higher estimates of Pb bioavailability were reported for liquids poor in nutritional elements (33-72%), and the lowest for milk (11-17%), traditional breakfast (0-8%), and calcium phytate (1-9%) [35]. Given the significant impact of food, the fasting state appears to be the conservative choice simulating the worst, but still plausible scenario, since soil, dust, and paint can be ingested by children during playtime between meals. However, Pb particles ingested via drinking water or formula, or incorporated into food during cooking can be ingested in both the fasting and the feeding state.

#### *In vivo* testing of Pb particles

Table 1 summarizes results from *in vivo* experiments completed with Pb-contaminated soils and Pb paint chips. Significant differences between the animal models, their age, the dosages, the duration, and the calculation method for generating RBA/ABA values must be taken into account when comparing these estimates. The extent of fasting is highly variable in these studies, and only a few studies were conducted in a complete fasting state [38,55,57,67,68].

In conclusion, several experimental factors should be considered, so that the results of *in vivo* Pb oral bioavailability estimates using animal models can be extrapolated appropriately to infants and children. In terms of the animal model, juvenile swine carefully controlled for age appear to be the best surrogate for human children. Of all the reported experiments and procedures, the conditions proposed by Casteel et al. [42,49], who used juvenile swine over a subchronic period of 15 days with 0.5-5 g soil (75–675 μg Pb/kg bw/day) delivered twice per day, appear the best suited for simulating the conditions of ingestion of soil/dust/paint particles by infants/children. The bioavailability calculation can be based on BLL estimates, or on a point estimate combining multiple IDMs, such as the widely used AUC for BLL, and the analysis of Pb accumulated in target tissues, such as bone, liver, and kidneys. Such an approach provides information on the distribution of body Pb and the interaction between accumulated lead in blood, soft tissues, and bones.

### *In vitro* bioaccessibility testing procedures

Bioaccessibility can be estimated at laboratory scale using chemical extractions of Pb contaminated test material in solution and under experimental conditions that mimic the mixing and processing of GI fluids. The total recoverable Pb is generally determined by subjecting a subsample of the test material [TM] to strong acid digestion, as, for example, in Method 3050 developed by the USEPA for soils and sediments. *In vitro* bioaccessibility [IVBA] is calculated as follows:

(6) $$\text{Pb}\;\text{IVBA}=\frac{\text{mg}\;\text{Pb}\;\text{leached}\;\text{in}\;\text{extraction}\;\text{fluid}\;\text{per}\;g\;\text{of}\;\text{TM}}{\text{mg}\;\text{total}\;\text{recoverable}\;\text{Pb}\;per\;g\;\text{of}\;\text{TM}}\text{.}$$

Several experimental factors affect the estimates of bioaccessibility yielded by *in vitro* tests, and their impact varies according to the type and form of the Pb sources investigated. Choosing an adequate combination of these factors is important for generating bioaccessibility results that can be correlated to bioavailability results. Key factors include the physico-chemical conditions maintained during the *in vitro* test, especially gastric and intestinal pH, but also the co-presence of food, mixing conditions, the solid to liquid [S/L] ratio, and the retention time. Their relative importance is summarized in Table 2. Finally, it should be noted that we discuss all the tests in this section, including those that were not correlated to *in vivo* data. In fact, although they were not calibrated, the latter tests provide an indication of the trends observed when the parameters of an *in vitro* test are varied, which makes it possible to rank the importance of each of these parameters.

**Table 2:** ***In vitro *tests design: main factors affecting bioaccessibility results**

**Parameters**	**Degree of Importance**	**General impact on IVBA%**	**Notes**	**References (non exhaustive)**
**Gastric pH**	+++	- IVBA ↑ when pH ↓	- Check the pH increase at the end of the G phase.	[69,81-84]
**Intestinal pH**	+++	- ↓ IVBA as compared to gastric IVBA	^-^ Adjust intestinal IVBA to Pb salt solubility during the intestinal phase	[65,67,85,86]
		- Major ↓ IVBA from pH 4 to 6		
			- Increase in solubility of bile/pancreatin-Pb complexes at pH 7.5	
		- Slight ↑ IVBA at pH 7.5 compared to pH 6.0-7.0		
**Temperature**	-	- No impact between 20°C and 37°C	- May be important when enzymes are added	[84]
**Phases simulated**	+++	- Gastric IVBA > Intestinal IVBA	- Adjust intestinal IVBA to Pb salt solubility during the intestinal phase	[67]
**Extraction**	+	- IVBA ↓ in the range: centrifugation > microfiltration > UF	- No difference between 0.2 and 0.45 μm filtration	[84,87]
**Fluid composition**	+	- Contradictory results (↑ or ↓ IVBA)	- Physiologically based fluids may be important when food is added	[55,67,85,88-90]
		- Physiologically based fluids do not seem important for the G phase		
			- Bile/pancreatin would create soluble complexes with Pb	
**Retention time**	++	- IVBA ↑ when gastric phase time ↑	- Lower impact on more soluble Pb forms: IVBA plateau reached after 20–30 min	[55,74,88,91]
		- No information on intestinal extraction time effect		
**Mixing**	++	- Aggressive mixing (Ar gas) ↑ IVBA	- End-over-end agitation adapted to maximize solid/fluid contact, but not too aggressive	[69,74,84]
**S/L ratio (g/mL)**	+++	- IVBA ↓ with high S/L > 1/100	- High S/L ↑ the effect of TM on pH ↑	[83,84,92]
		- No effect between low ratio 1/100 and 1/5000	- Low ratios (< 1/125) give poorer reproducibility	
**Food addition**	+++	- IVBA ↓ with food, except powdered milk	- Effect variable depending on food type	[62,87,88,93]
			- Fed tests linked to lower recovery rates	

**+++** high importance, **++** moderate importance, **+** light importance, **-** no importance, based on the studies published to date.

#### pH

Pb dissolution is very sensitive to pH. The correlation of *in vitro* to *in vivo* results for weanling rats, for example, has been shown to be critically dependent on the pH in gastric simulations [74]. The solubility of mineral species under the conditions that prevail in the stomach and upper intestinal tract determines their bioavailability, the factors affecting solubility being the mineral form, association, inclusion, and size distribution. Overall, chemical species formed under acidic conditions (e.g. Pb sulfate) will tend to be more stable in simulated acidic conditions and yield lower bioaccessibility values than those formed under alkaline conditions, such as Pb oxide and Pb carbonate [69]. Notwithstanding the specific dissolution rates of these forms of Pb, gastric pH stands out as a dominant factor influencing bioaccessibility results for a similar particle size distribution. For mine waste impacted soil and anglesite soil, Ruby et al. found that the rate of Pb dissolution was linearly dependent on HCl content during the first two hours of gastric retention [81]. Yang et al. measured Pb bioaccessibility values ranging from 50 to 80% for soil with a gastric pH of 2, those figures dropping to 10 to 20% at pH 3 or 4 [83]. Drexler and Brattin showed that pH was the most sensitive parameter in the RBALP: a variation in pH between 1.5 and 3.5 resulted in a variation in bioaccessibility by a factor from 2 to 7 that was only statistically significant above pH 2.5 [84]. Bruce et al. found significantly higher rates of gastric bioaccessibility for mine wastes tested at pH 1.3 (47%), as compared to pH 2.5 and 4 (17-18%) [94]. Oomen et al. concluded that gastric simulation pH was the major source of variability between the results of a round robin test of five *in vitro* models [82]. The stability of pH during the gastric phase also appears to be critical [95]. In fact, Oliver et al. found a higher bioaccessibility (26-46%) when the pH of the test was maintained at 1.3 than when the test was performed without any pH control (20-30%) [89]. Therefore, pH should be controlled at the end of the gastric phase, in order to provide the same acidic conditions for the various substrates tested. Results from the RBALP, RIVM, and UBM tests (calibrated) are only considered valid if the pH at the end of the experiment does not exceed a set reference value [66,84,86].

**Figure 3:**
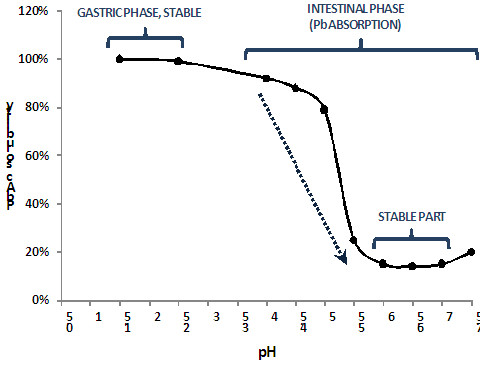
**Changes in Pb acetate (PbAc, 1-10 mg Pb/L) solubility with pH, during the gastric and intestinal phases. **Adapted from [67].

Juhasz et al. showed that the solubility of PbAc salt decreases greatly under simulated intestinal conditions (pH 4–7.5), reflecting the gradual increase in pH at the entrance to the intestine: from nearly 100% at pH 1.5, the IVBA decreases markedly between pH 4 and 6 to about 14.3±7.2% at pH 6–7 (Figure 3) [67]. Overall, initial concentrations in the range of 1 to 10 mg Pb/L did not influence solubility in the pH 1.5-7.5 range; slight differences were noted for high dosages of 5 and 10 mg/L with lower solubility at pH 5.5, and small but significant increases in solubility at pH 7.5. The steep decrease in PbAc solubility between pH 4.0 and 6.0 corresponds to the gradual increase in pH in the duodenum and jejunum, where most of the absorption and transport of Pb cations and complexes is supposed to take place [30,52]. Therefore, an intestinal phase carried out at pH 5.0 may not give the same results as an intestinal phase performed at pH 7.0. A realistic representation of the intestinal phase would include gradually increasing the pH and performing a series of sample collections during this increase, but doing so would be a huge challenge and would introduce much variability.

#### Temperature

The kinetics of Pb dissolution is known to be affected by temperature. However, for the conditions typical of *in vitro* experiments, temperature is not as strong a determinant of Pb dissolution as it is of the optimal activity of enzymes in the digestive juices. Drexler and Brattin found no significant difference between the RBALP results obtained at ambient temperature and those at human body temperature for seventeen test materials [84]. Most of the procedures apply a 37°C temperature by default, since temperature is a fixed parameter in the digestive process and closely mimics biological conditions.

#### Separation of the liquid from the solid

The separation method is an essential step in determining the bioaccessible Pb fraction [87], and the definition of the separation limit between particulate and dissolved Pb varies significantly in the literature and between models. Extraction can be achieved by centrifugation (e.g. [55,82,96]), centrifugation followed by filtration of the supernatant (e.g. [83,94]), or direct filtration (e.g. [84,89]). Others have used epithelial Caco-2 cells, in order to better represent the intestinal wall morphology [97]. Also, an aliquot of the digestion product can be analyzed directly, based on the hypothesis that the analytical instrument will only detect dissolved Pb [26,98].

In the absence of an extraction step, the digestion product should be analyzed immediately. Centrifugation can be applied at various combinations of rotational speed and time, which can influence separation and impact estimates of bioaccessible Pb. In filtration, the filter cut-off varies. Several studies used 0.45 μm filters [84,88,89], others used 0.2 μm filters [67,94], while the TIM model recommends ultra-filtration [UF] [82,87]. However, filtration at the end of the intestinal phase may bias results, since some filter materials are known to absorb dissolved Pb at neutral pH, the absorbed quantity varying with Pb concentration [99].

The impact of the various extraction methods identified above can be classified by the magnitude of bioaccessibility, as follows: no extraction > centrifugation > microfiltration > UF > Caco-2 cells. Van De Wiele et al. applied various extraction methods to the fed-RIVM, and bioaccessibility decreased from 31.5% (centrifugation) to 22% (microfiltration) to 3.5% (UF) [87]. Bioaccessibility results with UF were the closest to *in vivo* data on the same single soil tested [38,87]; however, the authors stress that these results are less reliable. Finally, Drexler and Brattin found no significant difference between bioaccessibility estimated with a filtration at 0.45 μm and one at 0.2 μm, suggesting that non bioaccessible Pb would be mainly particles > 0.45 μm for the test materials in question [84].

#### Fluid composition

The primary function of digestive enzymes is the decomposition of proteins (pepsin and trypsin) and carbohydrates (pancreatic and small intestine enzymes), while bile emulsifies lipids.

Oliver et al. showed that adding pepsin to the gastric fluid decreased the amount of dissolved Pb by about 28%. However, gastric pH did not remain constant, in fact, pH was increasing from 1.3 to 3 after the addition of the dust substrate, and this may be the factor responsible for the decreased bioaccessibility [89]. Conversely, Medlin compared bioaccessibility results using a physiologically-based gastric fluid and diluted HCl, and found little difference for cerussite, anglesite, and SRM 2710 at pH 1.5 [88]. However, at pH 2.8, the use of pepsin-based fluid increased bioaccessibility significantly (by up to 42%). Finally, Drexler and Brattin showed that the addition of enzymes was unnecessary for achieving a good *in vivo-in vitro* relationship using the validated RBALP procedure, which only simulates a gastric phase [84]. Consequently, for tests simulating this phase, the addition of enzymes appears unnecessary.

The simulation of physiological secretions may, however, be important for intestinal extraction. Ruby et al. found an increase in bioaccessibility of about 20% with the addition of organic acids and/or enzymes [55]. This was explained by the chelating characteristics of organic acids and by the binding of Pb to organic acids and enzymes, which kept the Pb dissolved in the intestinal fluid and prevented precipitation. Moreover, Juhasz et al. explained the increase in Pb solubility at pH 7.5 (compared to pH 6.0-7.0) by the formation of organo (pancreatin/bile)-Pb complexes [67]. Oomen et al. have suggested that the formation of Pb-bile complexes could increase Pb^2+^ concentrations [85]. In addition, the bioaccessibility results of Oomen et al. were about 10 to 40% higher for chicken bile than for ox or pig bile, which means that bile will affect bioaccessibility differently, depending on its animal origin [90]. Finally, in the case of an *in vitro* procedure simulating a fed state, the introduction of food may require the addition of enzymes and bile from both the gastric and intestinal phases, since they play a role in food degradation prior to absorption and in Pb complex formation [87].

#### Retention time

Retention times vary considerably between test methodologies and can impact the estimates of Pb bioaccessibility. Intuitively, for particles with limited solubility, longer stomach retention time should enhance bioaccessibility, since the extent of Pb dissolution increases with more contact time at a given rate. Ruby et al. evaluated Pb bioaccessibility in the stomach and intestine, and observed an average increase of 4.8% between 30 min and 1 h during the gastric phase, while an unsystematic decrease was noted during the intestinal phase [74]. Gasser et al. studied Pb dissolution in a stomach environment using a stirred flow reactor, and observed an initial rapid increase in Pb dissolution rate, which tended to decrease over time (0–60 min) and pH (1–3) [91]. Both the time and pH increment of this dissolution rate was substrate dependent. Moreover, Ruby et al. stated that, if the dissolution of the Pb-bearing mineral is controlled by a surface reaction rate, then the transit time will control that dissolution, suggesting that the impact of retention time in an *in vitro* test will depend on the form of the substrate studied [69]. For low solubility minerals, the dissolution kinetics would be controlled by surface reactions, and so the major parameter governing Pb release during digestion for these minerals would be stomach retention time. This is illustrated by Medlin’s results: less soluble Pb bearing minerals (anglesite, pyromorphite, galena) exhibited the highest dissolution rate, which continued to rise after the 1 h stomach simulation, whereas more soluble Pb ones (e.g. cerussite) generally reached a plateau after the first 20 min of the gastric simulation [88]. In another study, however, Drexler and Brattin applied 1, 2, and 4 h retention time to 11 substrates from comparable sources (mining sites) and found no significant differences [84].

#### Mixing

Determination of the mixing type and intensity depends on the test material studied. Ruby et al. stated that mixing controls the dissolution of Pb bearing materials through transport mechanisms [69]. For more soluble minerals, the kinetics of dissolution are controlled by transport mechanisms in which dissolved ions are quickly detached from the particle surface, accumulate, and form a saturation solution at the boundary layer. In addition, mixing conditions should be set to keep the Pb particles in suspension. Ruby et al. mixed by means of argon [Ar] injection, which efficiently kept the particles in suspension, but this type of aggressive mixing may overestimate bioaccessibility [74]. According to Drexler and Brattin, end-over-end mixing is the most appropriate for such experiments, because it maximizes the contact between sample and fluid (substrate surface area), and consequently Pb dissolution rate, and minimizes contamination caused by interacting devices (paddle, etc.) [84]. Moreover, it prevents the test material from sticking to the bottom of the tube/beaker, as observed for procedures using shaking water-bath (the authors’ observations in the laboratory).

#### Solid to liquid ratio [S/L]

The S/L ratio is another major factor impacting Pb dissolution and the resulting bioaccessibility estimation. According to Dean and Ma, the most common S/L ratios used for *in vitro* extractions vary between 1/2 and 1/250 g.mL^-1^[100]. Lower ratios, such as 1/1800 [101], 1/2160 [102], 1/5000 [92], and 1/16,667 [26], are also reported. The gastric ratio should be limited to reduce the effect of metal dissolution (observed for 1/5 and 1/25 ratios) and to prevent Pb chloride crystal precipitation upon cooling (for media containing over 50,000 mg Pb/kg) [84]. However, gastric ratios as high as 1/2.5 [82], 1/10 (e.g. [55,89]) and 1/30 to 1/40 have been applied (e.g. [103,104]). Such ratios may also increase the positive impact of the test material on the pH, if the latter is not controlled, and consequently underestimate bioaccessibility ( [86]). For the same BMW soil and similar PBET conditions at a 1/100 ratio, Ruby et al. found higher bioaccessibility (9.5-35%) than at a 1/10 ratio (≤ 6%) [74]. The 1/10 ratio used in Oliver et al. may have influenced the low bioaccessibility range found for dusts (20-30%), compared to other studies on dust for which Pb bioaccessibility generally exceeded 50% in the gastric phase [89]. Yang et al. demonstrated that Pb bioaccessibility in soil increased by about 10% between gastric ratios of 1/40 and 1/100 [83]. Hamel et al. had concluded that Pb bioaccessibility was only slightly affected with ratios between 1/100 and 1/5000 [92]. Medlin found that the Pb bioaccessibility of SRM 2710 soil increases by about 11% between the ratios 1/100 and 1/500 [88]. Drexler and Brattin standardized the procedure and recommended applying a ratio of 1 g of substrate per 100 mL fluid for media containing Pb in amounts less than 5% by weight (0.5/100 for substrates > 5 wt % Pb) [84]. Ratios below 1/125 are not recommended because of poorer reproducibility (substrate heterogeneity, weighing errors), as well as issues with the detection of low Pb concentrations during subsequent analysis. The effect of the S/L ratio during the intestinal phase has not been studied systematically, but could influence the precipitation reactions and also the stability of the solution, which has been shown to be difficult to maintain.

#### Food/substrate

The presence of food/substrate during digestion and its type will influence bioaccessibility, and a systematic decrease in bioaccessibility following the addition of food has been reported in most *in vitro* experiments, especially for gastric phase results. Indeed, dissolved Pb is adsorbed on non-digested elements, and forms complexes with some elements. Scheckel and Ryan found a Pb bioaccessibility close to zero for highly soluble hydrocerussite-based paint after the addition of a cola soft drink to the gastric fluid [93]. These results were explained by the reaction of soluble Pb with phosphates in the drink to form pyromorphite solid. Results obtained by Schroder et al. showed that the median bioaccessibility without dough added was 36.3% and 0.62% for the gastric and the intestinal phase respectively, while the test performed with dough resulted in bioaccessibility medians of 27% and 0.58% [70]. The swallow model performed by Brandon et al. in fasting conditions estimated the Pb bioaccessibility of ‘real-life’ paint at about 10%, while it was about 4.5% for the test performed with 4.5 g of infant food [101]. This decrease may be partly attributed to the difference in the stomach pH used to simulate a fasting state (1.6) or a fed state (2.5), but the presence of food may also have an influence. Additionally, the RIVM and TIM models run with and without solid food for the same soil resulted in higher bioaccessibility values for the fasting state (32-47%) than for the fed state (RIVM: 24-39%; TIM: 7%) [87]. Retention times and pH were quite close between the tests performed with and without food, suggesting that food would be partly responsible for this decrease. Recovery efficiencies influence these estimates, and the fed tests were linked to lower recoveries (70-93%) than were observed in fasting tests (≥ 83%) [87].

Remarkably, some studies performed with whole milk powder show the opposite trend, with an increase in bioaccessibility in a fed state. Marschner et al.’s two-phase enzymolyzis showed higher bioaccessibility for the fed state (11-56%) than for the fasting state (2-21%) [62]. With controlled retention time, pH, and fluid formulas, Van De Wiele et al. observed a higher bioaccessibility with the addition of whole milk powder to the fluid for the PBET and DIN tests (fed: 22-29%; fasting: 13-14%) [87]. Medlin investigated different types of food (banana, milk, oatmeal, and rice) and substrates (Pb phosphates, slag, galena, and Pb oxide) [88]. Trends varied depending on the food, the Pb form, and the amount tested, showing the huge challenges involved in the design of a representative fed state [84].

### *In vitro* testing bioaccessibility procedures and validation

Various methods have been published to investigate the bioaccessibility of Pb particles in environmental media, using simple to more complex methodologies aimed at reproducing to a varying extent the conditions in the digestive tract to which the particles are exposed. These methods can be classified in two categories:

1. Non physiologically based tests simulating the conditions of acidic pH and retention time in the stomach, without enzymes or other physiological fluids representative of real digestive conditions (or only additions that were shown to impact IVBA), namely: HCl extractions, the Relative Bioaccessibility Leaching Procedure [RBALP] or Simple Bioaccessibility Extraction Test [SBET], the Solubility Bioavailability Research Consortium procedure [SBRC], and variants of these tests.

2. Physiologically based extraction tests which attempt to simulate the physiological fluids and conditions of the digestive tract, namely: the Physiologically Based Extraction Test [PBET], the *In Vitro* Gastrointestinal method [IVG], the RIVM model, the Unified Barge Method [UBM], the DIN model, the TIM model, the SHIME model, and variants of these tests, either dynamic or static.

Table 3 summarizes the results obtained for the same test materials using various *in vitro* procedures. Depending on the procedure applied, the estimated IVBA varies widely, especially the intestinal IVBA. Results obtained with different *in vitro* test designs should be compared with caution, in light of the differences in testing conditions discussed previously. A more important aspect to consider is whether or not these tests provide estimates of bioaccessibility that are useful for refining bioavailability and Pb exposure assessments. In fact, the main objective of a bioaccessibility test is to offer a rapid and low cost alternative to bioavailability testing. Therefore, the optimal bioaccessibility test should produce data that can be adequately correlated to *in vivo* data. *In vivo* to *in vitro* correlation [IVIVC] can be used to calibrate *in vitro* testing conditions, which is highly desirable as it would ensure that an *in vitro* method is an acceptable alternative to animal investigations. These correlations are usually presented as linear regression models, and their strength will depend on the number of substrates tested and the range of IVBA/RBA available. According to Drexler and Brattin, a strong correlation is determined by an R^2^ > 0.6 and a significant relationship with a slope as close as possible to 1 [84]. However, it is suggested that the correlation coefficient may be more important than the slope, since a slope ≠ 1 does not impede linking IVBA to RBA [105].

**Table 3:** Comparison of bioaccessibility results obtained with the same test materials

**MATERIAL**	**TEST TYPE**	**GASTRIC IVBA %**	**INTESTINAL IVBA %**	**REFERENCES**
**Montana**	PBET	67.3		[88]
**SRM 2710 soil**	PBET	29±5 to 46±29		[92]
***RBA 76%***^***†***^	PBET		54-62±1^*^	[102]
	PBET	76.1±11	10.7±2.3	[57]
	PBET		30-35	[90]
	RIVM	79±4	25±1	[96]
	RBALP	75±5		[84]
	UBM	75	27	[65]
**Montana**	DIN		46±2	[82,90]
**SRM 2711 soil**	SHIME		3±0.3	
	TIM		17±3	
	RIVM		11±2	
	SBET	90±2		
	PBET		10-20 (60 for chicken bile)	
	PBET	85±5	13±1	[96]
	RBALP	84±6		[84]
	UBM	80	33	[65]
**Bunker Hill soil**	PBET	75		[88]
***RBAadults62±25%***^***‡***^	PBET		70±11^*^	[102]
	RIVM	87.6 ± 8.4	45.4 ± 4.0	[86]
	PBET		13±0.8	[87]
	DIN		13.6±0.6	
	SHIME		2.0±0.1	
	RIVM		31.8±2.5-47.4±3.2	
	TIM		32.5±4.5	
**Flanders soil**	DIN		31±3	[82]
	SHIME		4±1	
	TIM		13±3	
	RIVM		66±9	
	SBET	91±4		
**Oker 11 soil *RBA 55%***^***§***^	DIN		16±2	[82]
	SHIME		1±0.1	
	TIM		4±1	
	RIVM		29±2	
	SBET	56±4		
	IVG		20	[62]
	SBRC	66.8±2.3	62.9±11	[67]

IVBA–in vitro bioaccessibility;^*^bioaccessible Pb (mg) included Pb leached in G&I phases;^*†*^*RBA on juvenile swine*[65]*;*^*‡*^*RBA on humanadults*[38,86]*;*^*§*^*RBA on juvenile swine*[62].

Tests simulating a fasting state.

Another important aspect to consider in the selection of the *in vitro* testing procedure is the validation of the *in vitro* test. *In vitro* procedures should ideally be subjected to round robin testing to verify the ability of the results to be accurately reproduced or replicated. Inter-laboratory testing is needed to standardize the procedure, so that it can be applied in any laboratory on multiple test materials [65,84]. For example, the UBM procedure is considered valid when the relative standard deviation (RSD) of within-laboratory results and between-laboratory results was ≤ 10% and ≤ 20% respectively [95]. Then, multiple low cost experiments can be completed and extrapolated using existing data on animals and/or humans, and can be expected to be semi-quantitatively accurate. *In vitro* procedures that have not been calibrated or validated can nevertheless be useful, but should be considered as approximations and interpreted with caution.

#### *In vitro* test results compared to *in vivo* data

Table 4 lists *in vitro* tests that have been correlated to *in vivo* data, including variants of the PBET and IVG procedures: RIVM, UBM, RBALP, and SBRC.

**Table 4:** ***In vitro *procedures applied to Pb particles and compared with *in vivo *data for subsequent calibration (2 pages)**

**References**	**Substrate**	**Oral phase**	**Gastric phase**	**Intestinal phase**	**Comparison with *in vivo *data**
**Ruby et al. 1993***PBET-GI*[55]	Mine wastes		1/10 g.mL^-1^	2 h @ pH 7.0	· *In vivo* results on rabbits (1 sample): 10.7% (blood based RBA at t = 1 h), and 9^±4^ % (gastric solubility,t = 1.5 h)
			2 h @ pH 1.3 Pepsin; organic acids; HCl	NaHCO_3;_ pancreatin; bile	
					
**Ruby et al. 1996***PBET-GI*[74]	Mine wastes Residential soils Tailings		1/100 g.mL^-1^	4 h @ pH 7.0	· *In vivo* relative Pb bioavailability in rats (based on BLLs, Y axis) correlation to IVBA results from the G phase (X axis): R^2^ = 0.93, n=7, intercept 3.2, slope 1.4
			1 h @ pH 2.5 Pepsin; organic acids; HCl	NaHCO_3;_ pancreatin; bile	
					
					· Correlation with I phase: R^2^ = 0.76
**Medlin 1997** *PBET-G*[88]	15 soils or soil-like materials (EPA Region VIII)		1/111 g.mL^-1^		· *In vivo* point estimates in piglets (Y-axis) correlated to *in vitro* results (X-axis):
			1 h @ pH 1.5 Pepsin; organic acids; acetic acids; HCl		
					R^2^=0.63, n=15, intercept −8.21, slope 0.90, p < 0.001^*^
					· Extensive QA/QC protocol
**Brown et al. 2003***PBET-G*[58]	Urban soil treated with various biosolids (n=9)		1/100 g.mL^-1^		· *In vivo* bone bioavailability reduction % in rats correlated to *in vitro* bioaccessibility reduction with the treatment of soils (R=0.9)
			1 h @ pH 2.3 Pepsin; organic acids; HCl		
					
**Hettiarachchi et al. 2003***PBET-GI*[59]	Joplin soil treated or not with Mn, P, or CRYP (n=15)		1/100 g.mL^-1^	1 h @ pH 6.5	· *In vivo* point estimate RBA in rats (Y-axis) correlated to *in vitro* results (X-axis)
			1 h @ pH 2.0 Pepsin; organic acids; HCl	NaHCO_3;_ pancreatin; bile	
					G phase: R^2^=0.95, intercept 11, slope 0.82
					I phase: R^2^=0.77, intercept 12, slope 1.87
**Schroder et al. 2004***IVG*[70]	18 soils (EPA Region VIII)		1/150 g.mL^-1^ 1 h @ pH 1.8 Pepsin; NaCl; HCl	1 h @ pH 5.5 Pancreatin; bile; NaHCO_3_; decanol	· *In vivo* blood RBA (X-axis) in piglets correlated (p < 0.001) to *in vitro* data (Y-axis)^*†*^:
					- G (R=0.93) & I (R=0.80) results, with dough
					- G results no dough (R=0.89)
					· Best correlation for G phase with dough: R^2^=0.86, n=18, intercept 2.97, slope 0.39
					
**Marschner et al. 2006***IVG*[62]	5 soils, with or without milk powder		1/40 g.mL^-1^	6 h @ pH 7.5	· Absence of correlation between *in vivo* bioavailability in piglets and *in vitro* results
			2 h @ pH 2.0 Pepsin; NaCl; HCl	NaHCO_3;_ trypsin; bile; pancreatin; urea; inorganics	
					
**Oomen et al. 2006** *RIVM*[86]	11 soils or soil-like material (EPA Region VIII)	5 min @ pH 6.5	1/37.5 or 1/375 g.mL^-1^	2 h @ pH 5.5-6.5	· *In vivo* point estimates in piglets (X-axis) correlated to relative *in vitro* fasted model results (Y-axis):
			2 h @ pH 1.0-2.0	(in)organics, pancreatin, bile, BSA, lipase, CaCl_2_	G phase: R^2^=0.68-0.79, intercept 0, slope 0.79-1.08
		(in)organics, mucin, uric acid, alpha-amylase			
					I phase: R^2^=0.66-0.81, intercept 0, slope 0.69-1.16
			(in)organics, pepsin, mucin, BSA		
**Drexler and Brattin 2007; USEPA 2007***RBALP*[43,84]	19 soil-like materials from EPA region VIII,		1/100 g.mL^-1^		· *In vivo* RBA point estimates in piglets (Y-axis) correlated to *in vitro* results (X-axis) : weighted R^2^=0.924, n=19, intercept −0.028, slope 0.878, p < 0.001
			1 h @ pH 1.5		
			Glycine; HCl		
					· Extensive QA/QC protocol and statistical analyses
					· Precision 7% within samples, and 4-6% within laboratories (round robin testing)
**Bannon et al. 2009 ** *RBALP*[64]	8 small arms range soils		1/100 g.mL^-1^ 1 h @ pH 1.5		· *In vivo* point estimates on piglets: 108^±18^% · *In vitro* results: 83^±1^-100^±3^%
			Glycine; HCl		
**Caboche 2009Denys et al. 2012***UBM*[65,66]	14 mining and smelting soils	5 min @ pH 6.5	1/37.5 g.mL^-1^ 1 h @ pH 1.2-1.7	4 h @ pH 5.8-6.8	· *In vivo* kidney RBA in piglets (X-axis) correlated to *in vitro* results adjusted by PbAc solubility in the G and I phases (Y-axis):
				(in)organics, pancreatin, bile, BSA, lipase, CaCl_2_	
					G phase: R^2^=0.93, intercept 1.10, slope 1.86, p < 0.01
		(in)organics, mucin, uric acid, alpha-amylase			
					I phase: R^2^=0.89, intercept 1.09, slope 1.09, p < 0.01
			(in)organics, pepsin, mucin, BSA		
**Juhasz et al. 2009***SBRC*[67]	5 incinerator & urban soils; 5 soils from Marschner *et al.*[62]		1/100 g.mL^-1^ 1 h @ pH 1.5 Glycine, HCl	4 h @ pH 6.5NaOH, pancreatin, bile	· *In vivo* blood RBA in piglets (Y-axis) correlated to relative *in vitro* data for the I phase (X-axis): R^2^=0.53, intercept 1.98, slope 0.58 (urban & incinerator soils)
					R^2^=0.47, intercept 29.5, slope 0.42 (soils from Marschner et al., 2006 study)
**Wragg et al. 2011***UBM*[95]	12 soils (mining, composite, phosphate-treated), 1 dust	5 min @ pH 6.5 (in)organics, mucin, uric acid, alpha-amylase	1/100 g.mL^-1^ 2 h @ pH 1.2-1.4	4 h @ pH 6.3±0.5 (in)organics, pancreatin, bile, BSA, lipase, CaCl_2_	· *In vivo* RBA in piglets (X-axis) correlated to *in vitro* results adjusted by PbAc solubility in the G and I phases (Y-axis):
			(in)organics, pepsin, mucin, BSA		
					G phase: R^2^=0.61, slope 0.78, median RSD 4% within samples, 33% within laboratories
					I phase: R^2^=0.57, slope 0.38, median RSD 15% within samples, 81% within laboratories
**Smith et al. 2011***RBALP*[68]	12 soils impacted from a variety of Pb sources		1/100 g.mL^-1^ 1 h @ pH 1.5 Glycine, HCl		· *In vivo* blood RBA in mice (X-axis) correlated to relative *in vitro* data (Y-axis):
					R^2^=0.78, intercept 30.207, slope 0.69
**Smith et al. 2011***SBRC*[68]	12 soils impacted from a variety of Pb sources		1/100 g.mL^-1^ 1 h @ pH 1.5 Glycine, HCl	4 h @ pH 6.5 NaOH, pancreatin, bile	· *In vivo* blood RBA in mice (X-axis) correlated to relative *in vitro* data (Y-axis):
					R^2^=0.88, intercept −7.02, slope 1.06 (SBRC, I phase results)

IVBA–*in vitro* bioaccessibility; RBA–relative bioavailability; G–gastric; I–intestinal; RSD–relative standard deviation; ^*^relationship with corrected EPA *in vivo* data from [84]; ^†^*in vitro-in vivo* relationship not performed with EPA corrected data (*in vivo* bioavailability or bulk Pb concentrations).

#### PBET and IVG procedures

Numerous authors have used *in vitro* systems to estimate the bioavailability of inorganic elements. The PBET introduced by Ruby et al. [55,74] based on their previous work, was the first test for Pb particles that used simulated biological fluids in both the gastric and intestinal phases, and that correlated the results to *in vivo* data. As shown in Table 4, a synthetic gastric fluid composed of HCl, pepsin, and organic acids is added to the soil sample in a 1/100 g.mL^-1^ ratio. Initial incubation is performed at 37°C with varying initial pH (1.3, 2.5, 3.0, and 4.0), reflecting different digestion conditions under mixing with argon. After an hour, the pH is progressively adjusted to 7.0 using sodium bicarbonate [NaHCO_3_, simulated intestinal fluids (pancreatin and bile) are added, and mixing is continued for another four hours. The mixture is then centrifuged and settled, and the supernatant analyzed. A similar PBET test was developed by Medlin, using a comparable gastric S/L ratio and the similar synthetic gastric fluid formula [88]. The impact on the results of modifying the pH, time, particle size, and food addition, as well as the addition of a two-hour intestinal phase, was investigated. The final test applied only a gastric phase at pH 1.5 for 1 h, with continuous pH measurement, a mixing step combining Ar gas on the surface of the reaction vessel with the stirring rod moving at 60 rpm, and an extraction via 0.45 μm filtration. Brown et al. and Hettiarachchi et al. applied a similar version of PBET, as recommended by Ruby et al. [74], although the intestinal phase was either not simulated or faster (1 h) [58,59] (Table 4).

Results from the gastric phase using the Ruby et al. [74] procedure were calibrated for Pb in mine wastes with previous *in vivo* studies on Sprague Dawley rats. Only *in vitro* results of the gastric phase at pH 1.3 and 2.5 were linearly correlated to *in vivo* results (R^2^ = 0.93), with a slope closer to 1 at pH 2.5 [45,71-74]. The procedure by Medlin also showed that gastric bioaccessibility predicted *in vivo* juvenile swine data better than intestinal bioaccessibility (Table 4) [88]. Bioaccessibility estimates from the intestinal phase (tested for SRM 2710 and Pb oxide) underestimated the bioavailability data from *in vivo* calibration studies. Brown et al. observed a significant relationship between gastric RBA reduction and IVBA reduction following different Pb remediation methods performed on the tested soil [58]. Finally, Hettiarachchi et al. observed a stronger correlation of the RBA point estimates in rats with gastric IVBA, than with intestinal IVBA (Table 4) [59]. This lack of strong and significant correlations demonstrates the extent of the challenges in relating intestinal phase results from PBET to *in vivo* data, possibly due to precipitation reactions and the complexity of the mechanisms involved in intestine wall absorption.

Although the demonstration of a strong correlation with an animal model is highly valuable, the significance of the calibration is maximized if it is obtained using an adapted animal model that can be used to estimate exposure and risk assessment in human populations. The IVIVC observed for the PBET using rats [59,74], although significant, may not be as valuable for studying childhood exposure, however, because of the *in vivo* test design (animal model and/or dose administered), as underlined by Mushak [52]. In contrast, the PBET later developed by Medlin was correlated to *in vivo* data in juvenile swine [88], which are considered to be a better model for simulating digestion and absorption processes in children. In addition, this test was performed on a significant number of soils (n=15) using an extensive QA/QC protocol. Therefore, it appears to be more reliable than the Ruby et al. initial PBET [74]. Nonetheless, this test has not been subjected to the inter-laboratory validation and statistical analysis that would demonstrate good laboratory reproducibility of the results.

The IVG model, initially developed for PAH and PCB [106], was subsequently applied to Pb. It reproduces the PBET gastric and intestinal phases using physiological fluids, but the formulation of the fluids, retention time, and pH are different (Table 4). Schroder et al. and Marschner et al. compared the IVG results to *in vivo* results on swine using the same soils [62,70]. The absence of IVIVC in the Marschner et al. (2006) study was attributed to the technical difficulties during the intestinal phase simulation. However, Schroder et al. did find significant relationships between *in vivo* and *in vitro* results performed with and without dough: blood-based bioavailability was correlated to gastric *in vitro* results (R=0.93; p < 0.001) and to intestinal *in vitro* results (R=0.80; p < 0.001) with dough. *In vitro* tests performed without dough showed significant correlation to *in vivo* blood-based bioavailability in the gastric phase, but not in the intestinal phase (Table 4) [70]. However, the *in vivo* data used for this correlation were later corrected by Casteel et al. and the USEPA [42,43], and so the relationship would need to be re-evaluated with the corrected data prior to any use for other test materials. Finally, this test cannot be considered as a fully validated standardized *in vitro* procedure, because of the limited use of a QA/QC protocol and incomplete statistical analysis [84]. Nonetheless, it can be noted that, here again, the highest correlation was observed with *in vitro* gastric results.

#### RBALP and SBRC procedures

Considering the apparently good correlation of bioavailability and gastric bioaccessibility and the limited benefit of including an intestinal phase [88], a protocol reproducing only the gastric phase was developed in Dr. John Drexler’s laboratory. The RBALP was proposed after some investigation into the impact of temperature, contact time, pH, and S/L ratio to maximize correlation to *in vivo* data [84,88]. The RBALP includes an extraction with a synthetic fluid composed of glycine and HCl at 37°C (1 h, pH of 1.5, 1/100 g.mL^-1^). End-over-end mixing is performed (28±2 rpm), and separation is achieved via filtration at 0.45 μm. The test also includes criteria allowing for rigorous, reliable, and reproducible results that are only considered valid if: (1) the pH at the end of the extraction is within 0.5 units of the initial pH, which ensures that glycine buffering capacity is not exceeded; (2) the maximum holding time before analysis is one week; and (3) a rigorous QA/QC protocol is applied, including analysis of a bottle blank, a blank spike, a matrix spike, a duplicate sample, and a control soil at a 5-10% frequency. The test was applied to two SRM materials and nineteen test materials through round robin testing by four laboratories, and the results were compared via a statistical analysis. Intra-laboratory results showed good precision and agreement, with a CV of 2-6%, and the laboratory results respecting SRM standards were highly reproducible (CV of 7%). These observations confirm good reproducibility, precision, and standardization of the method.

As shown in Table 4, the RBALP was highly correlated to *in vivo* juvenile swine results using the nineteen test materials, including one NIST paint, galena, and soils impacted by mining and smelting activities [42,84]. Moreover, it is easier to perform than the PBET, because of the formulation of the synthetic fluids and the rapidity of the test. The British Geological Survey adapted the RBALP and renamed it the Simple Bioacessibility Extraction Test (SBET). The SBET protocol has been applied with some modifications [83,107]. Others applied the original RBALP [64,108,109]. The test applied to eight soils of small shooting ranges also revealed results comparable to an *in vivo* swine study (Table 4) [64]. Considering the number of substrates tested with the RBALP, which was calibrated with *in vivo* data on a recognized representative animal model and the results confirmed through a validation method, a rigorous QA/QC protocol, and round robin testing, this test stands out as a reference for application to other test materials. However, as underlined by the USEPA and Juhasz et al., although the substrates used for calibrating this procedure were numerous and originated from various sources, they do not represent the variety of Pb particles that exist in the environment. Also, they are made up of a significant number of substrates at mining sites for which Pb dissolution is limited during the gastric phase [43,67]. Therefore, the RBALP IVIVC may not hold for any other test material. Specifically, the RBALP applied to substrates containing more soluble forms of Pb than those found on mine sites, which partially precipitate/react during the intestinal phase, may overestimate Pb bioavailability [67]. Finally, results performed on soils following remediation with phosphates showed a better IVIVC with IVBA results performed at pH 2.5, suggesting that the RBALP would give more valuable results at pH 2.5 for evaluating soil remediation effectiveness [110,111].

To respond to these concerns, Juhasz et al. added an intestinal phase to the RBALP and compared the results obtained for the gastric and intestinal phases to *in vivo* bioavailability results on juvenile swine [67]. The SBRC intestinal test simulates a gastric phase identical to that of the RBALP, except that the agitation is performed at 40 rpm and the filtration on 0.2 μm filters, and then a 4-h intestinal phase is simulated at pH 6.5 with the addition of NaOH, bile, and pancreatin. In order to take into account precipitation reactions occurring during the intestinal phase and better relate intestinal IVBA to *in vivo* results, a relative intestinal IVBA was calculated. This was adjusted with the solubility of PbAc at pH 6.5 in the synthetic fluid, which was estimated at about 10-20%, as compared to 100% during the gastric phase at pH 1.5 (Equation 7). The application of an intestinal pH of 6.5 was justified by the fact that PbAc solubility was quite stable between 6.0 and 7.0, making the results more robust.

(7) $$\text{Relative}\;\text{Pb}\;\text{IVBA}=\frac{\frac{\text{in}\;\text{vitro}\;Pb_{\text{TM}}}{\text{total}\;\text{extractable}\;Pb_{\text{TM}}}}{\frac{\text{in}\;\text{vitro}\;Pb_{\text{PbAc}}}{\text{total}\;Pb_{\text{PbAc}}}}\text{.}$$

Gastric IVBA and absolute intestinal IVBA were poorly correlated to *in vivo* data for the five soils tested, while relative intestinal IVBA was satisfactorily correlated (R^2^ = 0.53). Although based on a weak fit (R^2^ = 0.12), and on few test materials, the authors state that the slightly negative Pearson correlation coefficient observed for the gastric phase may indicate an overestimation of the gastric phase estimates for some Pb forms. Applied to the same soils tested in the Marschner et al. study [62], relative intestinal IVBA estimates were well correlated to blood RBA on piglets, unlike the IVG results obtained by Marschner et al. [67]. This suggests that the calculation of relative bioaccessibility is a better RBA predictor. Interestingly, Smith et al. tested soils with a wide range of bioavailability (10-83%) and report excellent correlations between *in vivo* mouse data and both the RBALP test and the SBRC-intestinal test (Table 4) [68]. The slightly lower correlation obtained using the RBALP is explained by the overprediction of the RBA using this test for some soil types. This overprediction occurs because the presence of iron reduces the intestinal estimates, but not the gastric estimates, and so, the iron soil results correlated better using the SBRC-intestinal test [68]. It should be noted, however, that these conclusions were based on a comparison with mouse *in vivo* data, which is a less adequate animal model than the juvenile swine model. The IVIVC was then applied to the SBRC relative bioaccessibility results for thirty-one peri-urban soils, and compared to bioavailability predictions using the USEPA regression equation [43] on the SBRC results for the gastric phase. The bioavailability predictions were in agreement for shooting range soils, however different predictions were found for soils with high iron content, incinerator soils, historical fill soils, gasworks soils, and gossan soils (Fe-oxide rich soils). Overall, the RBALP relationships applied to the SBRC gastric results yielded more conservative bioavailability predictions than when the SBRC relationships were applied to the relative intestinal results [112]. This suggests that the addition of an intestinal phase to the RBALP, along with the use of relative bioaccessibility instead of absolute bioaccessibility, may yield bioaccessibility estimates that are closer to actual bioavailability results for some soil matrices. Nonetheless, as emphasized previously, additional inter-laboratory testing, statistical analysis, and further testing on juvenile swine (N=5) would be desirable to reinforce and standardize the SBRC-intestinal test, since, unlike the RBALP, this procedure has not yet been validated. We conclude that, while some refinements could be added to the RBALP, this test is the one that has been more thoroughly validated, and therefore is the most reliable.

#### RIVM and UBM procedures

The RIVM digestion model includes five minutes of simulated contact with saliva, followed by gastric and intestinal phases (two hours each) with end-over-end rotation at about 55 rpm in the presence of complex fluids. Simulation of the salivary phase is not generally added to *in vitro* models, because of the relatively short duration and neutral pH of this step. However, this step may be relevant when food is added, which is considered in the RIVM. For the test performed in the presence of food (macaroni, infant formula), adjustments are made in pH and fluid compositions to represent ‘stimulated’ conditions. Chyme pH is adjusted to 5.5-6.5 using NaHCO_3_ (Table 4). As for the RBALP, the test procedure is rigorously controlled for pH: 2.0 for the gastric phase, and 6.5 for the intestinal phase. Extraction is performed by centrifugation at 3,000 g and the pellet is kept for analysis and mass balance [82,86,87]. Bioaccessibility results for the fasting case were adjusted to PbAc solubility during the intestinal phase (relative IVBA) and compared to the USEPA data on juvenile swine on eleven soil-like materials. The relationship found was significant for the two S/L ratios tested (1/37.5 and 1/375 g.mL^-1^), for either the gastric or the intestinal phase [86]. The test was further applied in other studies [96,103]. Nonetheless, calibration of the RIVM was performed with bioavailability data from soils used for the RBALP calibration. It would be desirable to test the relationship with soils from more widely varied sources to verify this calibration.

The Unified BARGE Method (UBM) was developed by the Bioaccessibility Research Group Europe (BARGE) with the aim of selecting and standardizing a common procedure for bioaccessibility testing, for several trace elements (Pb, arsenic, cadmium, and antimony). In the UBM, which is similar overall to the RIVM, the retention time of the gastric phase is reduced to 1 h, and it is increased to 4 h for the intestinal phase [86]. IVBA results obtained with this approach for Pb were compared to *in vivo* RBA on juvenile swine for fourteen mining and smelting soils. Gastric and intestinal bioaccessibility showed strong IVIVC for Pb, arsenic and cadmium (Table 4) [65,66]. Therefore, the UBM could be applied for estimating the Pb, cadmium, and arsenic RBA for a single test material, and at the same time, which is an important advantage. This again suggests that the addition of the intestinal phase can be successfully linked to RBA results using the relative IVBA. However, in another study, results from the intestinal phase were not considered reliable for 13 other test materials, and those results did not meet any of the criteria set for validation (Table 4) [95]. Denys et al. concluded that the stomach phase of the UBM, carefully controlled for pH, produces IVBA results that are very comparable to juvenile swine RBA values [66]. Therefore, the addition of the intestinal phase, although possible, does not appear essential, and also complicates the test and its validation. The gastric phase of the test was validated for Pb, cadmium, and arsenic, and showed a significant IVIVC and reliable repeatability of the results [66]. An inter-laboratory trial was not able to fully validate the test for good reproducibility (between laboratories) for Pb. Nonetheless results suggest that a tight control of the gastric pH during extractions would allow for a complete validation [95].

The simulation of both the gastric and the intestinal phase greatly impacts the estimates of Pb bioaccessibility. Bruce et al. measured a bioaccessibility of 47% for the mine wastes at the end of the gastric phase, and these values dropped to 7% at the end of the intestinal phase [94]. The same range of decrease in bioaccessibility was observed in other studies [67,113-115]. Results using Pb-ISE and DPASV in chyme following GI *in vitro* digestion showed that the free Pb^2+^ is indeed negligible, and that most of the Pb was present as Pb-phosphate and Pb-bile complexes in dynamic equilibrium with solubilized Pb [85]. This solubilized Pb is largely removed from the solution by precipitation or adsorption on non-digested and compatible particles [67,74]. These observations show that Pb bioaccessibility could be overestimated if limited to the gastric phase. However, significant experimental challenges arise from the precipitation reactions, the gradual pH increase and parallel absorption, the eventual adsorption of soluble Pb on non-digested particles, and the increase of labile complexes that are difficult to maintain in equilibrium in the intestinal chyme [74,85]. Precipitation conditions may not be as well simulated in a closed system as in the gut, which is a thermodynamically open system [52], but spiking and correction for recovery could account for these losses. Comparison of Pb speciation by XANES after the gastric and intestinal phases has clearly shown the dominance of solubilized Pb after the gastric phase and the importance of the presence of co-precipitation with amorphous iron in soils containing iron oxyhydroxides [68]. To address some of these concerns, intestinal bioaccessibility can be adjusted relative to estimates of a soluble Pb form (e.g. PbAc), at a ‘stable’ intestinal pH (6.0-7.0) (Figure 3) [67]. With this adjustment, relative bioaccessibility estimates from the intestinal phase are slightly lower than the gastric bioaccessibility estimates, and seem to better relate to *in vivo* data [67]. However, results of tests simulating the gastric phase only are still more reliable, given that these tests are fully validated. Also, considering the difficulties associated with adding an intestinal phase, and although significant IVIVC can be obtained with intestinal results, full validation of the test with good repeatability and reproducibility would be quite challenging. To refine intestinal *in vitro* testing, further knowledge on the exact sites where Pb absorption takes place along the gut and at which pH would be necessary, so that the critical steps of the intestinal phase could be better reproduced.

In conclusion, the RBALP appears to be the best test yet devised for estimating the potential for childhood exposure to multiple Pb sources, even though it is not fully physiologically based. This test is the most suitable for the cost effective testing of multiple sources of Pb particles. It is likely to overestimate bioavailability for some test materials, and will produce a conservative IVBA estimate for these materials. However, the test is relatively simple and quick, and has been validated with *in vivo* data on the best suited animal surrogate for childhood digestive conditions. Moreover, it is the only procedure that has been validated through a complete statistical analysis and round robin testing. Consequently, the RBALP is standardized and can be applied in many laboratories on multiple test materials. The gastric phase of the UBM, which is currently in the process of being fully validated, is a pertinent alternative to the RBALP, as it tests the IVBA of three elements in the same procedure.

#### Tests not compared with *in vivo* data

Several *in vitro* tests that have not been compared with *in vivo* data have been proposed. These tests are important, since they may be validated in the future or be useful for studying factors influencing Pb bioaccessibility and bioavailability. Table 5 and Table 6 summarize the various parameters applied in some of these tests either not physiologically based (Table 5) or physiologically based (Table 6).

**Table 5:** **Examples of variants of non-physiologically-based *in vitro *procedures (not calibrated) applied to Pb particles**

**GENERAL**				**GASTRIC**			**INTESTINAL**	
**Reference**	**Mixing**	**T°C**	**Extraction**	**S/L (g.mL**^**-1**^**)**	**Time, pH**	**Fluid**	**Time, pH**	**Fluid**
**Sheppard et al. 1995**[116]			Filtration 0.5 μm	1/167	24 h, pH 2.0	HCl		
**Gasser et al. 1996**[91]	Stirred-flow reactor	24°C	Filtration 0.22 μm	1/100	1 h, pH 1.0 to 3.0	HCl, ammonium acetate		
**Gasser et al. 1996**[91]	Horizontal shaker	24°C	Centrifugation (400 RCF; 10 min)	1/200	1 h, pH 1.0	HCl		
**Rieuwerts et al. 2000**[117]	Inversion by hand 5 times at t = 1h	Ambient	Centrifugation (2,000 rpm; 2 min)	1/100	2 h, pH 1.2	HCl		
**Yang et al. 2001**[118]	Rotation 30 rpm	37°C	Filtration 0.2 μm	1/100	1 h, pH 2–2.5	HCl		
**Mercier et al. 2002**[119]	End-over-end 30 rpm, at 20 min intervals, for 20 min	35-39°C	Decantation, filtration	1/22	160 ±10 min, pH 6.0 to 2.0	HCl, acetic acid		
**Yang et al. 2002**[120]	Rotation 30 rpm	37°C	Filtration 0.2 μm	1/100	1 h, pH 2.0	HCl		
**Scheckel and Ryan 2003**[93]	Continuous stirring	37°C	Filtration 0.45 μm	1/160	1 h, pH 2.0	HCl	5 h, pH 7.0	
**Yang et al. 2003**[83]	End-over-end,30 rpm	37°C	Decantation, filtration 0.45 μm	1/40 and 1/100	1 h, pH 1.5 to 4.0	HCl, glycine	3 h, pH 7.0	NaHCO_3_
**Beak et al. 2006**[121]	Variable speed mixer (150 rpm); 2 mL/min Ar	37°C	Filtration 0.45 μm	1/167	2 h, pH 1.8	HCl	4 h, pH 7.0	NaOH
**Turner and Simmonds 2006**[98]		Ambient		1/100	Overnight	HCl, pepsin		
**Bosso and Enzweiler 2008**[107]	Orbital, 100 rpm	37°C	Centrifugation (5,000 rpm; 20 min)	1/100	1 h, pH 1.5	HCl, glycine OR HCl, pepsin, NaCl	2 h, pH 7.0	NaHCO_3_
**Le Bot et al. 2010, 2011**[122,123]	Ultrasonication	37°C	Filtration 0.45 μm		1 h, pH 1.5	HCl , 0.75-1.4% diluted		
**Rasmussen et al. 2011**[124]		37.5°C	Centrifugation (5,000 g, ≤ 10 min)	1/2000	2 h, pH 1.5	HCl		

**Table 6:** **Examples of variants of physiologically based *in vitro *procedures (not calibrated) applied to Pb particles (2 pages)**

**GENERAL**			**ORAL**		**GASTRIC**			**INTESTINAL**	
**References**	**Mixing**	**Extraction**	**Time, pH**	**Fluid**	**S/L (g.mL**^**-1**^**)**	**Time, pH**	**Fluid**	**Time, pH**	**Fluid**
**Sheppard et al. 1995**[116]		Centrifugation, filtration 0.2 μm				4 h, pH 2.0	NaCl; pepsin; HCl	18 h, pH 7.5	NaHCO_3_; NaCl; bile; pancreatin; α-amylase
**Berti and Cunningham 1997**[125]	Stir bar				1/100	1 h, pH 2.5	Pepsin; organic acids; HCl	2 h, pH 7.0	NaHCO_3_; pancreatin; bile
**Davis et al. 1997**[126]	Wrist action shaker	Centrifugation (2,100 g; 25 min)			1/10	2 h, pH 1.3	Pepsin; organic acids; HCl	2 h, pH 7.0	NaHCO_3_; pancreatin; bile
**Hamel et al. 1998**[92]	Shaking water bath	Centrifugation (1,380 g; 10 min)			1/100 to 1/5000	2 h	NaCl; pepsin; HCl		
**Hamel et al. 1999**[102]	Water bath, 90 cyc/min	Centrifugation (906 g; 10 min), filtration 0.45 μm	5 s, pH 5.5	Mucin; urea; KCl; NaCl; Na_2_HPO_4_; CaCl_2_.4H_2_O	1/2160	2 h	NaCl; pepsin; HCl	2 h	NaHCO_3_
**Oliver et al. 1999**[89]	Wrist action shaker	Filtration 0.45 μm			1/10	2 h, pH 1.3 to 3.0	Organic acids; HCl; with(out) pepsin	16 h, pH 7.0	NaHCO_3_ with(out) bile & pancreatin
**Ellickson et al. 2001**[57]	Bath set, 90 cyc/min	Centrifugation (200 g; 20 min) & (906 g; 15 min); HNO_3_, 48 h; filtration 0.45 μm		Mucin; urea; CaCl_2_.H_2_O; NaCl; KCl; Na_2_HPO_4_	1/2160	2 h, pH 1.4	Pepsin; NaCl; HCl	4 h, pH 6.5	NaHCO_3_
**Oomen et al. 2002, Van De Wiele et al. 2007**[82,87]; *DIN test*	Agitator, 200 rpm	Centrifugation (7,000 g; 10 min)			1/50	2 h, pH 2.0	HCl; [pepsin; mucin]^*^	6 h, pH 7.5	Phosphate buffer; [bile; trypsin; pancreatin]^*^
**Oomen et al. 2002, Van De Wiele et al. 2007**[82,87]; *SHIME test*	Stirring, 150 rpm	Centrifugation (7,000 g; 10 min)			1/2.5 to 1/40	3 h, pH 2.0 (fast) or 4.0 (fed)	Nutrilon plus; pectin; mucin; cellobiose; proteose peptone; starch; glucose	5 h, pH 6.5	NaHCO_3_; pancreatin; bile
**Oomen et al. 2002, Van De Wiele et al. 2007**[82,87]; *TIM test*	Peristaltic	Ultrafiltration	5 min, pH 5.0			Fast: 40 min, pH 4.5 to 1.8	HCl; lipase; pepsin^*^ 0.5 mL/min	Fast: 5.3 h, pH 6.5 to 7.2	NaHCO_3_; pancreatin; bile^*^ 1 mL/min
								Fed: 4.5 h, pH 6.5 to 7.2	
						Fed: 1.5 h, pH 5.0 to 2.0			
**Yu et al. 2006**[113]	Water bath, 90 rpm	Filtration 0.45 μm		Mucin; urea; KH_2_PO_4_; CaCl_2_.H_2_O; NaCl, KCl	1/400	2 h, pH 1.4	NaCl; pepsin; HCl	2 h, pH 6.5	NaHCO_3_
**Bruce et al. 2007**[94]	Ar	Centrifugation (10,000 g; 15 min), filtration 0.22 μm			1/100	1 h pH 1.3 (fast) to 4 (fed)	Pepsin; HCl; organic acids	3 h pH 7.0	NaHCO_3_; bile; pancreatin
**Saikat et al. 2007**[104]		Centrifugation (2,100 g; 25 min)			1/38-1/100	1 h pH 1.1 or 2.5	Pepsin; organic acids; HCl	4 h, pH 5.5 or 7.0	NaHCO_3_; pancreatin; bile
**Triantafyllidou et al. 2007**[26]	Gentle mixing	No separation			1/16,667	3 h, pH 1.2	NaCl; pepsin; HCl		
**Turner and Ip 2007**[127]	End-over-end	Centrifugation (2,100 g;10 min)			1/200	2 h, pH 2.5	Pepsin; HCl; Na malate & citrate; lactic & acetic acids	4 h, pH 7.0	NaHCO_3_; bile; pancreatin
**Van De Wiele et al. 2007**[87]	Water rotator set	Filtration 0.45 μm			1/100	1 h pH 2.5	Pepsin; HCl; Na citrate & malate; lactate, acetate	4 h, pH 7.0	NaHCO_3;_ bile; pancreatin
**Bosso and Enzweiler 2008; Bosso et al. 2008**[107,114]	Slow orbital + Ar flux	Centrifugation (5,000 rpm; 20 min)			1/100	1 h, pH 1.7	Pepsin; HCl; citric, malic, acetic, & lactic acids	2 h, pH 7.0	NaHCO_3;_ bile; pancreatin
**Turner et al. 2009**[128]	Constant, lateral	Centrifugation (2,000 g; 10 min)			1/100-1/143	1 h, pH 2.5	Pepsin; HCl; Na malate, citrate; lactic & acetic acids	4 h, pH 7.0	NaHCO_3_; bile; pancreatin
**Sialelli et al. 2010**[115]	Orbital, 150 rpm				1/100	1 h, pH 1.5	Pepsin; HCl; Na citrate; malic & lactic acids	3.5 h, pH 7.0	Pancreatin, NaHCO_3_

Procedures that are not physiologically based (Table 5) include a large number of batch acid extractions using dilute HCl performed at various S/L ratios, durations, and pH. Since 1994, HCl extraction has been a standard method for assessing the toxicity of toys used to establish the European Standard on the Safety of Toys. The toy material is reduced to a particle size < 500 μm and added to an HCl solution using a 1/50 g.mL^-1^ ratio (pH 1.5; 37°C; 2 h) [50,129]. As pointed out by Le Bot et al. [122], such tests may be helpful in preventing Pb poisoning. In fact, Pb leached during HCl extractions simulating stomach conditions may provide a more relevant indication of potentially bioavailable Pb than standard total Pb extractable measurements on wastes and solids. Some sources could have high values of total extractable Pb even though it is poorly soluble in the stomach, or, inversely, they could impair the interpretation of BLLs and environmental Pb levels.

Among the physiologically based models presented in Table 6, many are modifications of the PBET, IVG, or RIVM procedure. The German method, E DIN 19738 [DIN] mimics gastrointestinal digestion for infants/children, in a fasting case or a fed state, through the addition of whole milk powder. Gastric fluids are composed of a mixture of pepsin, mucin, and HCl. Intestinal fluids contain bile, trypsin, pancreatin, and phosphate buffer. The gastric phase takes place at pH 2 over 2 hours, and a pH of 7.5 is applied to the intestinal phase over 6 hours. Extraction is achieved by a two phase centrifugation at 7,000 g, decantation, and analysis of the supernatant [82,87]. Although somewhat laborious, DIN appears to be a relevant method, as there is a need for a test with whole milk powder for infants exposed to Pb particles in drinking water via baby feeding bottles.

The TIM, SHIME, and flow-through methods using online extractions are dynamic, physiologically based tests [87,130,131]. The TIM model (or TNO GI model) mimics the contact with saliva and further GI digestion by the progressive addition of fluids, the progressive adjustment of pH, and the simulation of peristaltic contractions. Gastric pH and retention times are adjusted differently if the simulation is aimed at representing a fed state (pH decreases gradually from 5 to 2 over 90 min) or a fasting state (pH decreases gradually from 4.5 to 1.8 over 40 min). Then, intestinal fluids are added at 1 mL/min to increase the pH from 6.5 to 7.2, at which point ultra-filtration is performed. This model has been validated with *in vivo* dissolution profiles of drugs with or without food; however, it was not calibrated for Pb particles [82,86,87]. The SHIME (Simulator of Human Intestinal Microbial Ecosystem of Infants) model, developed in Belgium, simulates 3 h gastric and 5 h intestinal digestion in the same reactor (150 rpm). Gradients of pH can be applied if a series of reactors is used (dynamic model), and a phase including a mixed microbial community can be added to the model [82,132]. Constant stomach retention times are applied for fasting conditions (pH ~ 2.0) and fed conditions (pH ~ 4.0) [87]. Centrifugation at 7,000 g is conducted and the supernatant is analyzed. The pellet is then digested to mass balance [82]. However, these procedures cannot be used routinely to measure Pb bioaccessibility on Pb particles since they take too long to perform and are highly complex. Nevertheless, it appears that they could be highly useful for studying specific factors affecting Pb bioavailability that have been poorly studied up to now, such as the effect of bacteria.

### Bioavailability/bioaccessibility of Pb particles in relation to Pb speciation, particle size, and surrounding matrix

Physical and chemical aspects of Pb particles are major factors influencing the dissolution of particles containing Pb. Table 7 summarizes the IVBA results for calibrated procedures, and shows that bioavailability and bioaccessibility results vary widely with the test material: from 1.7-6% for galena to 100% for shooting range soil, which is in agreement with the *in vivo* swine data [42,64,68,84,86], and from 1.5 to 100% for tap water particles [29]. Pb was also found to be highly bioaccessible in NIST Paint (75-86%), which is in agreement with previous epidemiological studies referenced in Mushak [30]. In addition, Pb is generally highly bioaccessible in smelter soils, with about 68-69% for some Omaha community soils, and about 70-85% for the Herculaneum smelter [61,63], and 34-90% for ten smelting soils from northern France [65]. However, variability in the bioavailability and bioaccessibility data can be noted for certain substrates, which reflects the highly heterogeneous nature of these substrates in terms of Pb form, particle size, etc. The bioaccessibility of three slag materials was evaluated at 17, 20, and 73% [84]. Also, among mining site soils and urban topsoils, the IVBA was shown to vary from low (9-14%) to high (63-75%) [65].

**Table 7:** **Main bioaccessibility results for materials tested using an *in vitro *procedure calibrated with *in vivo *data**

**SUBSTRATE**	**IVBA%**	**REFERENCES**
Flanders soil	91±4	[29,43,60,61,63,64,82,84,109]*RBALP (Gastric)*
Oker 11 soil	56±4	
47 Omaha community smelter soils, US	68-69 (average)	
HER-2930 smelter soil, US	69±1.5 (using Dr Drexler’s Pb levels)	
	85±1.1 (using EPA’s average bulk Pb levels)	
17 residential soils, tailings, and slags from mining waste sites, US	14±1.7 to 90±3.1	
1 NIST paint	75±3.8	
1 Galena	6±2.3	
8 shooting range soils, US	83±1 to 100±3	
20 soils from the N-S transect, US (agricultural, grazing land, open range, forested land, residential, desert)	3.7 to 39	
70 samples of tap water particles	1.5 to 100	
9 soils from mining sites*	3.1±0.1 to 99.3±14.3	[86]*RIVM (Intestinal)*
1 NIST paint* + soil	86.2 ± 2.3	
1 Galena*	1.7 ± 0.2	
15 mining soils, France	9-75 (Intestinal)	[65,66,95,133,134]*UBM*
10 smelting soils, France	34-90 (Intestinal)	
27 urban topsoils, France	11-63 (Intestinal)	
12 soils (mining, composite, phosphate-treated), 1 dust, from various studies (European and North American)	0.6±0.1 to 112.8±18.5 (Gastric)	
	0.1±0.1 to 89.5±91.3 (Intestinal)	
2 urban residential soils, Australia	20.0±4.0 to 26.1±6.5	[67,68,112]*SBRC (Intestinal)*
3 domestic incinerator soils, Australia	11.7±2.8 to 22.5±5.0	
	3.2±2.6 to 8.5±0.6^*†*^	
Brushal, Carl-1, Hamburg, and Oker-11 soils^§^	30.7±6.1 to 62.9±11.0	
9 shooting range soils, Australia	21.3-102.6	
	59±3.7 to 92±9.0 (3 soils)^*†*^	
5 historical fill soils, Australia	5.5-26.1 (4 soils)	
	10.7±0.7 to 16.6±1.1 (2 soils)^*†*^	
13 mining/smelting soils, Australia	11.6-82.5	
	31±18.4 to 74±17.3 (3 soils)^*†*^	
1 gasworks, Australia	27.2	
	27±1.6^*†*^	
1 geogenic, Australia	12.5	

^*^Same test materials as in RBALP studies, 0.06 g;^*§*^*soils tested by Marschner et al.*[62]*;*^*†*^*Smith, et al.*[68].

#### Pb speciation

Speciation in the particles can be a strong indicator of the potential bioavailability of Pb. Indeed, RBA estimates can vary widely (6-105%) for Pb particles across the same site or region (e.g. California Gulch, CO), reflecting variability in their mineral composition and the potential of Pb to be liberated [42]. According to Schoof et al., the solubility of Pb minerals present in the test material explains the observed differences in bioavailability results [40]. In addition, Rasmussen et al. successfully predicted their bioaccessibility results on dust from 924 Canadian homes by identifying the Pb phases present with XANES, and further cumulating the bioaccessibility specific to each phase [124]. Identifying the major species present in a soil would therefore help in predicting the extent of bioavailability. Schoof et al. classified the range of solubility of Pb minerals as very high for Pb oxide [PbO]; average for Pb-manganese [Pb-Mn] oxides, Pb-iron [Pb-Fe] oxides, and Pb carbonates; moderate for Pb sulfates and Pb arsenate; and minimal for Pb phosphates [Pb-P] [40]. The reference USEPA classification for bioavailability (and therefore bioaccessibility) after several *in vivo* and *in vitro* studies varies from high for cerussite [PbCO_3_ and Pb-Mn oxide (> 75%), to medium for Pb-P and PbO (25-75%), to low for anglesite, galena, Fe-Pb species and remaining Pb-based oxides (< 25%) [43]. Drexler and Brattin observed the lowest bioaccessibility for substrates containing anglesite or galena as dominant Pb species (6-21%) [84]. On the contrary, substrates with either PbCO_3_ or MnOOH as the dominant Pb mineral systematically presented a high bioaccessibility (65-90%), in agreement with the data on juvenile swine. Pb carbonate species are easily soluble, and this is reflected in other *in vitro* results: Pb carbonates were estimated to be 97% bioaccessible in the Schaider et al. study [108]; hydrocerussite paint 69% bioaccessible prior to cola addition in another study [93]; and Pb was 56% (gastric) and 25% (intestinal) bioaccessible in the high carbonate garden soil G1 in the Denys et al. study [96].

Soil remediation with phosphates has been shown to decrease Pb bioaccessibility, in agreement with the expected low solubility of Pb-phosphate particles [114,118,120,135]. Nonetheless, a low bioavailability/bioaccessibility fraction should not be interpreted as representing a low hazard potential. Indeed, in the Schroder et al. study [70], the bioavailability of Pb in the Pb-rich soil 9 (10,600 mg Pb/kg soil) is medium to low (RBA 20%, ABA ~ 10%), but a dose of 100 mg of soil ingested daily will release the same quantity in the body, about 0.1 mg of Pb, like the highly Pb-bioavailable but less Pb-rich soil 15 (RBA 74%, ABA ~ 37%, 3230 mg Pb/kg soil) (authors’ calculation). Another facet of speciation concerns the major impact of the rate of pH change on the solution and mineralogical composition found at a given pH. Marked differences in solubility were shown between tests conducted in dynamic versus static pH adjustment conditions for a soil dominated by cerussite and modified with hydroxyapatite [135]. The differences reached up to three orders of magnitude in the presence of phosphates, and were attributed to the mechanisms that govern the amount of soluble Pb, with adsorption/desorption phenomena decreasing the amount of soluble Pb in dynamic systems.

#### Particle size

It is generally accepted that a small particle size provides a high surface to volume ratio and an elevated potential for dissolution [69]. Indeed, Casteel et al. found a high RBA (57-58%) for soils of ≤ 250 μm containing a majority of particles ≤ 10 μm [49]. The significant BLLs observed in piglets dosed with “predicted low bioavailable” tailings were partly explained by the small size of the galena crystals (mostly < 10 μm) that were completely dissolved within 50–100 min at a low gastric pH [79]. Rieuwerts et al. reported a bioaccessibility of about 61-116% for particles < 64 μm, while particles > 64 μm were 21 to 72% bioaccessible [117]. Mercier et al. showed that Pb bioaccessibility generally decreased with increasing granulometry (< 63 μm to 125–250 μm), but remained stable or varied without any trend between 125–250 μm and < 2 mm, depending on the substrate [119]. Yu et al. [113] did not find any significant differences in the bioaccessibility estimates between the dust size fractions < 75 μm, 75–150 μm, and 150–250 μm, and, finally, Morman et al. [109] found similar ranges for soil particles < 2 mm (8.5-77%) as compared to particles < 250 μm (3.7-45%), suggesting that small particles are not always more bioaccessible. Such differences may be partly explained by the Pb phase of the particles tested: less soluble Pb phases (anglesite, pyromorphite, and galena) were more influenced by particle size than the more soluble Pb phases in the Medlin study [88], bioaccessibility increasing about 4- to 13-fold between the fractions < 38 μm and < 250–125 μm. Moreover, Ruby et al. showed that Pb dissolution rates are not affected by particle size, except for diameters less than 2.4 μm [81]. Finally, Oliver et al. noted that greater bioaccessibility for small particles is more evident for equivalent diameters below 100 μm, so the impact of particle size on bioaccessibility results would only be significant for very small and colloidal particles [89].

For a given test material, an increase in bioaccessibility/bioavailability that is in inverse proportion to particle size may reflect a relative enrichment in smaller size fractions. Indeed, Juhasz et al. report up to five times more Pb in the < 50 μm particle size fraction of sixteen peri-urban soils [136]. Also noted were significant increases in gastric-SBRC IVBA for six of those soils, but no differences in intestinal-SBRC IVBA between the size fractions. Madrid et al. observed a higher Pb content in the clay fraction (< 2 μm) of urban soils from Sevilla, as well as an increase in the bioaccessible Pb in this size fraction [137]. Finally, a 110% average Pb enrichment was measured in the particle size fraction adhering to hands for different types of soils in Canada, as compared to the bulk Pb content [138]. As the fraction adhering to hands is usually smaller than the < 250 μm particle size fraction traditionally used for soils, these authors recommend conducting bioaccessibility experiments on small particles, for example < 45 μm.

#### Matrix characteristics

The characteristics of the matrix surrounding Pb can also influence Pb release for a given type of particle. Pb bioaccessibility has been related to total Pb content in soils, but also to other metal levels in the soils tested, such as zinc, iron, and cadmium [133,139]. Strongly positive linear relationships were found between Pb bioaccessibility and Pb content in the soils [133]. It can be expected that a higher Pb content will result in greater bioaccessibility. However, Morman et al. found no correlation between total elemental content (Pb, Cd, Ni, Cr, and As) and the bioaccessible fraction for the twenty soils from various sources [109]. Comparable RBA results (56-58%) were obtained with Aspen Berm and residential soils of comparable particle size, matrix, and mineral type, although the Berm soil contained four times as much Pb [49]. Oomen et al. also found higher bioaccessibility for Flanders sandy loam (91%) than for Oker 11 sandy loam (56%), although the latter contained ten times more Pb [82]. This indicates that bioavailability is more influenced by the particle type distribution than by its Pb content.

Bioaccessible Pb was related to other characteristics of the soil matrix than total metal content, such as carbonates, clay, and organic matter, in soils from similar sources [133,140]. However, another study did not find a significant relationship between Pb bioaccessibility and the total amount of carbonates in a variety of high Pb-carbonate soils [96]. Caboche et al. found significant correlation to the cation exchange capacity; organic matter; and clay, manganese, phosphorus, and iron content for soils from the same source. However, no correlations were found when soils from all the sources tested were pooled [134]. In addition, the bioaccessibility of twenty soils from various sources in another study was not correlated to the organic carbon, pH, and clay percentage in the soils [109]. These findings suggest that significant relationships with soil characteristics can be found for soils from the same source type, but that they cannot be generalized to other matrices.

Overall, soil characteristics will impact Pb bioaccessibility differently, depending on the Pb species present, and cannot be used as a general predictor of bioaccessibility, while Pb speciation may be more predictive of bioaccessibility.

### Gaps in bioaccessibility data

This review documented an abundance of peer-reviewed studies on *in vitro* testing, on topics ranging from simple leaching procedures to validated procedures calibrated to *in vivo* animal studies, and their application to a range of Pb bearing substrates. Among all the methods developed to estimate Pb bioaccessibility, only a few have been successfully compared with *in vivo* data (Table 4), and only the RBALP has been calibrated, validated, and fully standardized [84]. Other procedures, such as UBM, RIVM, and SBRC, have been highly correlated to *in vivo* data, and sometimes nearly fully validated (UBM), and so their results can also be considered valid for improving exposure assessment and public health protection. However, additional validation with statistical analysis and round robin testing will be needed to standardize these procedures.

A significant number of substrates were tested using these validated procedures (Table 7). However, few data (one) are available on bioaccessibility on dust, although dust Pb content was evaluated at some of the sites studied. Also, only one paint substrate was tested and the NIST material used may not necessarily be representative of paint chips to which children are exposed. However, paint containing Pb is not unusual in toys and old buildings [141], and recent results on paint chips using HCl extraction show high variability of leachable Pb, ranging from 4 to 100% [122]. As well, some HCl extractions performed on dust particles have shown that the amount of leachable Pb is generally high [126], and this is partly attributed to the small size of these particles. Pb dust and Pb paint have been shown to be major contributors to the BLLs of children [142]. Further research is needed to quantify the variability in bioaccessibility for these particles, to support the estimation of the contribution of paint and dust particles to exposure, and to help analyze the reported pica-caused BLL cases associated with ingestion of these particles. There is also a significant data gap regarding Pb particles from drinking water systems. Early results using the RBALP adapted for Pb particles from tap water show that the bioaccessibility of particles generated from plumbing metals and collected from distribution systems varies widely [29]. Such data are needed, considering that: (i) particulate Pb can be sporadically high and is currently not fully considered in drinking water sampling and analysis methods; (ii) baby bottles may be prepared with tap water; and (iii) tap water Pb particles and colloids are small and may be highly soluble, depending on the forms present [26,27,143].

Finally, most of the bioaccessibility results were measured in a fasting state, which is considered to be the worst case ingestion scenario. This ingestion state may be realistic for soil, paint, and dust particles, but not for tap water Pb particles, which can be ingested in either the fasting or the fed state. The oral bioavailability of Pb in food cooked or prepared with high Pb particulate water might also be a relevant route of exposure to study [26]. However, the development and validation of a standardized *in vitro* test for a fed state would certainly be a challenge, considering the variability of food that can be ingested by children (other than milk) and the divergent results observed for several types of food [88]. The most disturbing finding is that higher bioaccessibility values are found for *in vitro* tests performed with whole milk powder than for those simulating a fasting state [62,87]. In fact, early studies show that milk consumption increases the absorption of PbAc in rats [144]. This has also been observed for hydrophobic organic compounds, and can be explained by the formation of soluble metal complexes with milk constituents [86,106]. Such information may not have been considered in the past because the major sources of Pb exposure were air and soil, and Pb exposure from these sources is not likely to occur at the same time as milk ingestion. However, considering that water is now established as a significant residual source of Pb exposure and that baby bottles are often prepared with tap water, the ingestion of formula made in this way probably constitutes the worst case exposure, as it involves both the dissolved and the particulate Pb in tap water. Therefore, it is not clear that *in vitro* procedures in a fasting state will provide worst case assessments of exposure for bottle-fed infants/children. One *in vivo* study was performed with the simultaneous dosage of soil and milk powder on juvenile swine [62]. However, the dosage of soil was not tested on its own, precluding any comparison between Pb absorption from soil particles alone and Pb absorption from particles in the presence of milk. The apparent increase in Pb bioavailability when Pb is ingested with milk certainly needs to be confirmed with an *in vivo* test on piglets, to compare both soluble and particulate Pb ingestion, with and without milk.

## Conclusion

*In vivo* experiments on Pb particles have been carried out using animal models on a wide range of particulate Pb forms, mostly from contaminated soils. It is established that there are substantial anatomical and physiological differences between animal species and humans, especially children. The scarcity of data providing a direct estimate of the human absorption of Pb particles is a significant limitation on our ability to estimate bioavailability, a shortcoming that is partially addressed by the use of the most representative animal model. Of all the animal models evaluated, juvenile swine are considered the most appropriate animal model for human exposure studies. However, results from animal models should always be considered with caution when extrapolated to humans or used to validate results from *in vitro* testing. Critical factors to consider in order to ensure relevance to public health decision making include: (1) the limitations and specific features of the animal model; (2) targeting the human population in the design of animal studies, specifically the appropriate developmental stage; and (3) the use of plausible environmental doses and Pb speciation.

Results reporting Pb bioaccessibility reflect the experimental conditions considered, and, in the absence of a standard procedure, cannot be compared. At the same time, this variability is inextricably linked to the natural variability of human exposure to Pb particulates. The RBALP procedure seems well suited to Pb particles, since its results for gastric extraction can be successfully correlated to *in vivo* data on piglets. This test was completely validated and submitted to a rigorous QA/QC protocol. The addition of an intestinal bioaccessibility phase to better mimic Pb solubility at the neutral pH of absorption (UBM, SBRC, RIVM) may refine the bioavailability estimates. However, these tests are more costly than the RBALP and quite tedious, and the full validation of the tests simulating an intestinal phase is likely to be extremely challenging. The “stomach” UBM, which is in the process to be fully validated, appears to be a reliable test, and offers the major advantage of estimating the RBA of two other elements as well as Pb (arsenic and cadmium).

Finally, the validated tests were applied on a significant number of substrates, but mostly soils. The selection does not cover the whole variety of possible matrices surrounding Pb in environmental sources and other significant sources of exposure, such as those of paint, as well as dust and tap water. Testing these types of particles *in vitro* raises experimental challenges, because of their heterogeneity and the small amounts of some of them, but is needed to complete the input in exposure models and risk assessment studies. The RBALP could be adapted for estimating bioaccessibility from these sources, since this test is quite simple to perform compared to other procedures, and presents the highest degree of standardization.

## Competing interests

The authors declare they have no competing interests.

## Authors’ contribution

ED and MP carried out the literature research for the review and drafted the manuscript. RT, ME, and SS reviewed and completed the manuscript. All authors read and approved the final manuscript.
